# *Chaetomella raphigera* β-glucosidase D2-BGL has intriguing structural features and a high substrate affinity that renders it an efficient cellulase supplement for lignocellulosic biomass hydrolysis

**DOI:** 10.1186/s13068-019-1599-0

**Published:** 2019-11-02

**Authors:** Mu-Rong Kao, Hsion-Wen Kuo, Cheng-Chung Lee, Kuan-Ying Huang, Ting-Yen Huang, Chen-Wei Li, C. Will Chen, Andrew H. -J. Wang, Su-May Yu, Tuan-Hua David Ho

**Affiliations:** 1 Molecular and Cell Biology, Taiwan International Graduate Program, Academia Sinica and National Defense Medical Center, Taipei, Taiwan, ROC; 2 Institute of Molecular Biology, Academia Sinica, Taipei, Taiwan, ROC; 3 Institute of Plant and Microbial Biology, Academia Sinica, Taipei, Taiwan, ROC; 4 Department of Environmental Science and Engineering, Tunghai University, Taichung, Taiwan, ROC; 5 Institute of Biological Chemistry, Academia Sinica, Taipei, Taiwan, ROC; 6 Department of Bioengineering, Tatung University, Taipei, Taiwan, ROC; 7 Biotechnology Center, National Chung Hsing University, Taichung, Taiwan, ROC

**Keywords:** *Chaetomella raphigera*, GH3, β-Glucosidase, Saccharification, *O*-Glycosylation, Lignocellulosic biomass

## Abstract

**Background:**

To produce second-generation biofuels, enzymatic catalysis is required to convert cellulose from lignocellulosic biomass into fermentable sugars. β-Glucosidases finalize the process by hydrolyzing cellobiose into glucose, so the efficiency of cellulose hydrolysis largely depends on the quantity and quality of these enzymes used during saccharification. Accordingly, to reduce biofuel production costs, new microbial strains are needed that can produce highly efficient enzymes on a large scale.

**Results:**

We heterologously expressed the fungal β-glucosidase D2-BGL from a Taiwanese indigenous fungus *Chaetomella raphigera* in *Pichia pastoris* for constitutive production by fermentation. Recombinant D2-BGL presented significantly higher substrate affinity than the commercial β-glucosidase Novozyme 188 (N188; *K*_m_ = 0.2 vs 2.14 mM for *p*-nitrophenyl β-d-glucopyranoside and 0.96 vs 2.38 mM for cellobiose). When combined with RUT-C30 cellulases, it hydrolyzed acid-pretreated lignocellulosic biomasses more efficiently than the commercial cellulase mixture CTec3. The extent of conversion from cellulose to glucose was 83% for sugarcane bagasse and 63% for rice straws. Compared to N188, use of D2-BGL halved the time necessary to produce maximal levels of ethanol by a semi-simultaneous saccharification and fermentation process. We upscaled production of recombinant D2-BGL to 33.6 U/mL within 15 days using a 1-ton bioreactor. Crystal structure analysis revealed that D2-BGL belongs to glycoside hydrolase (GH) family 3. Removing the N-glycosylation N68 or O-glycosylation T431 residues by site-directed mutagenesis negatively affected enzyme production in *P. pastoris*. The F256 substrate-binding residue in D2-BGL is located in a shorter loop surrounding the active site pocket relative to that of *Aspergillus* β-glucosidases, and this short loop is responsible for its high substrate affinity toward cellobiose.

**Conclusions:**

D2-BGL is an efficient supplement for lignocellulosic biomass saccharification, and we upscaled production of this enzyme using a 1-ton bioreactor. Enzyme production could be further improved using optimized fermentation, which could reduce biofuel production costs. Our structure analysis of D2-BGL offers new insights into GH3 β-glucosidases, which will be useful for strain improvements via a structure-based mutagenesis approach.

## Background

Lignocellulosic biofuels are considered environmentally friendly sources of transportation energy to replace fossil fuels and food-based biofuels [1]. Production of lignocellulosic biofuels involves using agricultural waste as feedstock, which avoids the air pollution generated by waste biomass incineration and decomposition. Combustion of bioethanol releases water and carbon dioxide, which can be recaptured by plants to generate new biomass, so cellulosic biofuels are considered carbon neutral.

Recent research has aimed to improve the quality of lignocellulosic feedstock, pretreatment processes, and the efficiency of enzymatic hydrolysis to reduce biofuel production costs [2, 3]. Continuing efforts to discover new cellulolytic enzymes and to optimize enzyme production are necessary to make lignocellulosic biofuels as economically competitive as classical fuels.

Synergy among three types of cellulases is essential for the complete hydrolysis of cellulose, which represents 23–60% of dry weight in lignocellulose [4, 5]. Endo-glucanases (EC 3.2.1.4) break down internal 1,4 β-glycosidic bonds in the cellulose chain, and exo-glucanases (including β-1,4-glucan cellobiohydrolase EC 3.2.1.91 and 1,4-β-d-glucan glucohydrolase EC 3.2.1.74) digest the chain endings to release cellobiose [6]. β-Glucosidases (EC 3.2.1.21) finalize the cellulolytic process by hydrolyzing cellobiose into glucose. During cellulose saccharification, large amounts of β-glucosidases must be added to avoid feedback inhibition of exo-glucanases and endo-glucanases by cellobiose and to compensate for the loss of β-glucosidase catalysis efficiency due to accumulation of both cellobiose and glucose [7]. Therefore, β-glucosidase efficiency is evaluated by three criteria: dynamic synergism with the other two cellulases, catalysis efficiency of cellobiose, and tolerance to substrate and product inhibition.

Fungal β-glucosidases are widely used in the biofuel industry because of their high catalytic efficiency of cellobiose and cello-oligomers. *Aspergillus niger* β-glucosidase Novozyme 188 (N188) is the commercial enzyme most widely applied in biofuel production. This enzyme has the ability to hydrolyze lignocellulosic biomass efficiently in combination with *Trichoderma reesei* cellulases, so it is frequently used as a reference in studies of β-glucosidases [8, 9]. *T. reesei* expressing heterologous *Aspergillus aculeatus* β-glucosidase AaBGL1 exhibits twofold greater saccharification efficiency of NaOH-pretreated rice straws than the wild-type strain [10]. Mutants of *Aspergillus* species β-glucosidases with enhanced catalysis efficiency have been generated using random mutagenesis [11, 12]. Enzymes from other filamentous fungi have also been characterized for their high thermal and pH stability [13–16] or tolerance to glucose inhibition [17], which are specific features necessary for industrial applications.

Many high-efficiency β-glucosidases have been discovered in the last decade, but two major issues must be overcome before commercialization. Native fungi have a low level of enzyme production, so efficient expression systems with suitable post-translational glycosylation are required for large-scale production of recombinant fungal enzymes. In addition, due to the importance of fungal β-glucosidases in industrial applications, several 3D crystal structures of GH3 (glycoside hydrolase family 3) enzymes from *Aspergillus* and other fungal species have been studied [18–21]. However, revealing specific structural features of enzymes with high cellulolytic activities shall facilitate the improvement of GH3 β-glucosidases via structure-based mutagenesis.

D2-BGL is a GH3 β-glucosidase from the indigenous Taiwanese fungus *Chaetomella raphigera*. Our research team previously demonstrated that native D2-BGL works in dynamic synergism with *T. reesei* cellulases to effect cellulose hydrolysis [22]. In the present study, we performed molecular and biochemical characterizations of recombinant D2-BGL expressed by *Pichia pastoris*, as well as crystal structure analysis. We also investigated biomass hydrolysis and ethanol production by this enzyme via a semi-simultaneous saccharification and fermentation process, and conducted large-scale production of our recombinant D2-BGL in a 1-ton bioreactor to evaluate its potential for commercialization.

## Methods

### Culture of *C. raphigera* D2 strain

The D2 strain of the fungus *C. raphigera*, which was isolated from the gut of the endemic Taiwanese termite *Odontotermes formosanus*, was maintained on potato dextrose agar (PDA; Difco, BD). It was cultured by transferring a plug of 7-day-old mycelia (~ 5 mm^*3*^) onto a newly prepared plate. For on-plate enzyme assays, 1 g/L esculin and 0.3 g/L ferric citrate were added to PDA medium before autoclaving, and the medium was then inoculated with fungus. β-Glucosidase activity was observed by the formation of dark esculetin–Fe^*3*+^ complex. For flask assays, actively growing hyphae were inoculated into 100 mL of fresh Mandels–Reese medium [23] containing 1 g/L soy peptone, 1.4 g/L (NH_4_)_2_SO_4_, 0.3 g/L urea, 2 g/L KH_2_PO_4_, 0.34 g/L CaCl_2_, 0.3 g/L MgSO_4_·7H_2_O, 5 mg/L FeSO_4_·7H_2_O, 1.6 mg/L MnSO_4_·7H_2_O, 1.4 mg/L ZnSO_4_·7H_2_O, 2 mg/L CoCl_2_·6H_2_O, and 0.72 g/L cellobiose in a 250-mL flask. After 4-day incubation at 30 °C with shaking at 125 rpm, the culture broth was collected for enzyme activity analysis.

### Phylogenetic and protein structure analyses

Evolutionary analysis of GH3 enzymes was performed using Molecular Evolutionary Genetics Analysis version 7.0 (MEGA7) software, and a phylogenetic tree was built by the neighbor-joining method [24, 25]. Structure alignment and visualization of protein 3D crystal structures were carried out using the PyMOL Molecular Graphics System v2.2.3 (Schrödinger, LLC). Multiple alignment of protein sequences was performed using Clustal Omega [26].

### Heterologous expression of D2-BGL in *P. pastoris*

Cloning of D2-BGL cDNA into pGAPZαC vector (Invitrogen, USA) was performed as described [27] to generate the expression vector *Pp* D2-BGL #1. To increase purification yield by affinity chromatography, a second vector (*Pp* D2-BGL #5) was generated by inserting an additional 6-histidine tag at the N terminus of D2-BGL. We performed codon optimization by substituting the nine “CGC” triplets coding for arginine by “AGA” to increase enzyme production. Transformation of *P. pastoris* strain SMD1168 via electroporation was performed according to the user manual in the *Pichia* Expression Kit (Invitrogen). Transformants were selected on YPDS plates (1% yeast extract, 2% peptone, 2% glucose and 1 M sorbitol) with 100 mg/L Zeocin (InvivoGen).

### Sample preparation before purification

To purify D2-BGL, *P. pastoris* cells were removed from the fermentation broth by centrifugation at 10,000*g* for 10 min. The supernatant was successively filtered through 0.8-µm, 0.45-µm and then 0.2-µm Supor Membrane Disc Filters (PALL). Concentration and buffer exchange of the filtered solution were performed at 4 °C using Hollow Fiber Cartridge Model UFP-10-E-3MA Type 10.000 NMWC (GE, USA) according to the user’s manual. For the buffer exchange, 1 L of crude enzyme solution was concentrated to 200 mL. The concentrated solution was diluted to 400 mL with phosphate binding buffer (20 mM sodium phosphate and 500 mM NaCl, pH 7.4). The diluted solution was again concentrated to 200 mL and the dilution/concentration process was repeated three times.

For Novozyme 188 (Sigma), 1 mL of commercial solution was diluted in 50 mL Tris buffer (50 mM Tris and 150 mM NaCl, pH 7). The enzyme solution was filtered through a 0.45-µm Supor Membrane Disc Filter (PALL) and concentrated to 1 mL with Amicon Ultra-15 30 K NMWL (Millipore). Buffer exchange was performed twice with 15 mL Tris buffer. The final sample for purification was made up to 50 mL with Tris buffer.

### Purification of β-glucosidase D2-BGL and Novozyme 188

Purification was carried out by means of an ÄKTA-FPLC automatic liquid chromatography system (GE). D2-BGL was purified by immobilized metal affinity chromatography (IMAC) with a HisPrep FF 16/10 20-mL column (GE). The column was equilibrated with five column volumes (CV) of binding buffer (20 mM sodium phosphate and 500 mM NaCl, pH 7.4). After injection of 50 mL of enzyme sample, the column was washed with 5 CV of binding buffer. Elution was conducted using a total volume of 20 CV, with a linear gradient from 0 to 60% of elution buffer (20 mM sodium phosphate, 500 mM NaCl and 500 mM imidazole, pH 7.4).

Novozyme 188 (N188) was purified in two steps. In the first step, it was partially purified by anion-exchange chromatography using a HiTrap Q HP 5-mL column (GE). The column was equilibrated with 5 CV of starting buffer (50 mM Tris, pH 8). After injection of 50 mL of sample solution, the column was washed with 5 CV of starting buffer. Elution was performed with a total volume of 20 CV, with a linear gradient from 0 to 60% of elution buffer (50 mM Tris and 1 M NaCl, pH 8). Purity of elution fractions with high *p*NPGase activity was verified by a strong band in SDS-PAGE. Fractions showing β-glucosidase activity were collected, buffer exchanged and then concentrated to 1 mL with 50 mM Tris and 150 mM NaCl, pH 7. The second step of purification was performed by size-exclusion chromatography using a HiPrep Sephacryl S-100 HP column (GE). Protein content and *p*NPGase activity were measured for each elution fraction. Fractions with high specific activity were collected and concentrated using Amicon Ultra-15 10 K NMWL (Millipore). After buffer exchange with Tris buffer (50 mM Tris and 137 mM NaCl, pH 7), the purified enzyme was stored at 4 °C.

### Cellulase activity assays

β-Glucosidase activity was assayed with *p*-nitrophenyl β-d-glucopyranoside (*p*NPG) or cellobiose as substrate in sodium acetate buffer (NaOAc 50 mM, pH5) at 55 °C. For the *p*NPGase assays, a standard curve was established from OD_405_ values of serial dilutions of *p*NP from 1.25 to 0.078 mM. We mixed 100 μL of enzyme solution in a 1.5-mL Eppendorf tube with 100 μL of 4 mM *p*NPG. After 5 min, 600 μL of 1 M Na_2_CO_3_ was added to stop the enzyme reaction. The OD_405_ value was measured with 200 μL of final reaction solution in a 96-well plate using a SpectraMax M2e microtiter plate reader (Molecular Devices, USA). For cellobiase assays, 100 μL enzyme solution was mixed with 100 μL of 20 mM cellobiose, and the reaction was stopped by heating at 100 °C for 10 min. Glucose content was measured using a YSI 2700 Select Biochemistry Analyzer (Yellow Springs Instruments, USA). One enzyme unit (U) was defined as 1 μmol of product released per minute.

Exo-glucanase, endo-glucanase, total cellulase and xylanase activities were determined using Avicel, carboxymethylcellulose (CMC), Whatman no. 1 filter paper or xylan as substrates, respectively. The enzyme unit, defined as 1 μ mol of reducing sugars released per minute, was determined according to the dinitrosalicylic acid (DNS) method [28].

### Effects of temperature and pH

To determine pH and thermostability, enzyme solutions containing 1.2 μg of purified enzyme were incubated for 4 h at different temperatures or for 24 h at different pH. To establish optimum temperature and optimum pH, 0.03 μg of purified enzyme was used to perform *p*NPGase assay for 5 min at different temperatures and pH.

### Enzyme kinetics

Enzyme activity assays were performed at 55 °C for 10 min. Activities were determined using 0.03 μg β-glucosidase at different *p*NPG (0.25 to 14 mM) or cellobiose (0.625 to 40 mM) concentrations. Kinetic parameters *K*_m_, *V*_max_ and *K*_i glucose_ were determined by non-linear curve-fitting in Prism 8 (GraphPad Software Inc., USA). The effect of glucose inhibition was determined in the presence of 0, 10 and 20 mM glucose.

### Biomass hydrolysis assays

The estimated cellulose content of pretreated rice straws and bagasse, provided by the Institute of Nuclear Energy Research (INER, Taiwan), was 44.7% and 48.17% (w/w), respectively. Pretreated biomass was dried in an oven at 60 °C to remove residual moisture, and then the dried material was ground into fine powder (estimated diameter: < 0.5 mm).

The *T. reesei* RUT-C30 cellulase mixture and commercial enzyme mixture CTec3 were provided by INER. The commercial *T. reesei* cellulase mixture Celluclast 1.5L (C1.5L) was obtained from Sigma. Avicel and β-glucosidase activities were determined for all tested cellulase mixtures to ensure that saccharification efficiency was limited by β-glucosidase activity in each mixture.

The activity of different enzyme combinations was tested with 1% (w/v) rice straw or sugarcane bagasse powder in 1 mL sodium succinate buffer (50 mM at pH 5). The hydrolysis reaction was performed at 50 °C for 24 h. Glucose contents were measured using a YSI 2700 Select Biochemistry Analyzer.

### Semi-simultaneous saccharification and fermentation process

The semi-simultaneous saccharification and fermentation (SSSF) process was performed in a 100-L bioreactor containing 20% w/v acid-pretreated rice straws in 50 mM sodium acetate buffer, pH 5. For the acid pretreatment, chopped rice straws (6–15 mm length) were soaked in an acid solution containing 0.5–3% sulfuric acid. After transferring the mixture into a filter bag, liquid was removed by filtration under pressure at 8 MPa for 5 min. The remaining biomass was treated by steam explosion (150–200 °C). An enzyme solution was prepared by mixing ten volumes of *T. reesei* cellulases (20 FPU/mL) with one volume of β-glucosidase N188 (263 U/mL *p*NPGase activity) or D2-BGL (88 U/mL *p*NPGase activity). The resulting enzyme solution was adjusted to 15 FPU per gram of glucans.

To prepare the *Saccharomyces cerevisiae* culture, single yeast colony was pre-cultured in 5 mL of YPD medium at 30 °C and 150 rpm for overnight. The overnight culture was inoculated into 50 mL YPD medium in a 250-mL flask for 24 h. The 50-mL pre-culture was inoculated into 7 L YPD medium in a 10-L bioreactor for another 24 h to obtain the yeast culture with an OD_600_ value reached to 15 to 20.

SSSF began with a pre-saccharification phase in 63 L of fermentation solution containing 14 kg of acid-pretreated rice straw, 4.7 L of *T. reesei* cellulases (20 FPU/mL) and 0.47 L of β-glucosidase (263 U/mL for N188 or 88 U/mL for D2-BGL) at 50 °C for 12 h, followed by the simultaneous saccharification and fermentation phases at 33 °C for a further 72 h after inoculation with 7 L of the aforementioned *S. cerevisiae* culture.

### Production of D2-BGL by fermentation in a 1-ton bioreactor

The fermentation medium was prepared with glycerol (40 g/L), trace element solution (1 mL/L) and a salt solution (0.38 g/L CaCl_2_, 18.2 g/L K_2_SO_4_, 9.4 g/L MgSO_4_·7H_2_O, 4 g/L KH_2_PO_4_ and 4 g/L (NH_4_)_2_SO_4_). The trace element solution contained 2.5 g/L MnSO_4_·H_2_O, 54.17 g/L FeSO_4_·7H_2_O, 16.67 g/L ZnCl_2_·2H_2_O, 0.17 g/L Na_2_MoO_4_·2H_2_O and 19.2 mL/L H_2_SO_4_ (96.2%). The salt solution and glycerol were sterilized by autoclaving, and the trace element solution was sterilized by filtration. The feeding solution, which was added continuously to supply nutrients for yeast growth during the fermentation process, was prepared with 2 g/L (NH_4_)_3_PO_4_, 600 g/L glycerol, 1 g/L glucose and 1 mL/L trace element solution.

One colony of *P. pastoris* expressing D2-BGL was pre-cultured in 10 mL of YPD medium at 30 °C and 150 rpm. After 24 h, 5 mL pre-culture was inoculated into 250 mL YPD medium for another 24 h to obtain the seed culture. Fermentation started with inoculation of 250 mL seed culture into the 5-L bioreactor containing 4 L of fermentation medium and proceeded at 30 °C with air flow at two vessel volumes per minute (vvm), 20% dissolved oxygen (DO), 600 rpm and pH 5.5. The pH was adjusted with 5 N H_2_SO_4_ as acid and 30% ammonia as base. After 24 h, the entire fermentation broth was transferred into a 100-L bioreactor containing 50 L fermentation medium, and the fermentation continued at 30 °C with air flow 2 vvm, 20% DO, 150 rpm and pH 5.3–5.7 for 30 h. Upon adding the resulting high cell density culture prepared from the 100-L bioreactor into 400 L of fermentation medium, fermentation was performed in the 1-ton bioreactor at 30 °C with air flow 2 vvm, > 20% DO, 50 rpm and pH 5.2–5.8. When the glycerol had been totally consumed and the DO reached ~ 50%, fermentation medium was added every 25 min for 40 s at 650 mL/min. The temperature in the bioreactor was decreased to 25 °C when the yeast culture reached 40 g dried cell weight (DCW)/L. Supplementary trace element solution was added (1 mL/L) every 100 h.

### Crystallization and data collection

D2-BGL crystals were grown by mixing 1 µL protein (10 mg/mL) with 1 µL reservoir solution and using the sitting-drop vapor diffusion method at 18 °C. The crystals were obtained in a reservoir solution of 10% (w/v) PEG 3000, 0.2 M magnesium chloride, 0.1 M sodium cacodylate, pH 6.5. D2-BGL crystals were flash-cooled with 20% glycerol (v/v) as a cryo-protectant. The diffraction data were collected at cryogenic temperatures at wavelength 1.000 Å on a beamline BL12B2 of the Spring-8 synchrotron in Japan, with a Quantum-210 CCD detector. All diffraction data were processed and scaled using the program HKL2000 [29].

### Structure determination and refinement

D2-BGL crystal structures were determined by molecular replacement using the program MOLREP of the CCP4 program suite [30], and the crystal structure of β-glucosidase 1 (PDB: 4IIB) from *A. aculeatus* [18] was used as a search model. The D2-BGL crystal belongs to space group P2_1_2_1_2_1_. Throughout the refinement, 5% of randomly selected data were set aside for cross validation with Rfree values. Manual modifications of the models were performed using the program Coot [31]. Difference Fourier (Fo–Fc) maps were calculated to locate the solvent molecules. Crystal structures were refined using Refmac5 [32]. The molecular figures were generated in UCSF Chimera [33]. The atomic coordinate and structure factor of D2-BGL have been deposited in the Protein Data Bank (accession code 6JXG).

### Construction of D2-BGL mutants by site-directed mutagenesis

D2-BGL mutants were generated using a QuikChange II Site-Directed Mutagenesis Kit (Agilent Technology, USA). Primer design and PCR conditions were determined as recommended in the user manual. Briefly, 20 ng plasmid was used as a template for PCR amplification with back-to-back primers. PCR conditions were initial denaturation at 95 °C for 2 min, 18 cycles of denaturation at 95 °C for 30 s followed by annealing at 60 °C for 30 s and extension at 68 °C for 3 min, and a final extension at 68 °C for 5 min. The PCR product was treated for 1 h at 37 °C with the restriction enzyme DpnI before transformation into *Escherichia coli* strain DH5α.

### Deglycosylation with endoglycosidase H and peptide *N*-glycosidase F

D2-BGL was deglycosylated using endoglycosidase H (Endo H, NEB) or peptide *N*-glycosidase F (PNGase F, NEB) to remove N-linked glycans. Briefly, 20 μg enzyme was treated under denaturing (i.e., by heating in deglycosylation buffer at 100 °C for 10 min) or non-denaturing (i.e., without heating) conditions. To test residual activity after non-denaturing treatment, cellobiase assays were performed with 0.006 μg of deglycosylated enzyme and 10 mM cellobiose at 55 °C for 20 min.

## Results and discussion

### Expression of *C. raphigera* β-glucosidase D2-BGL in *P. pastoris*

**Fig. 1:**
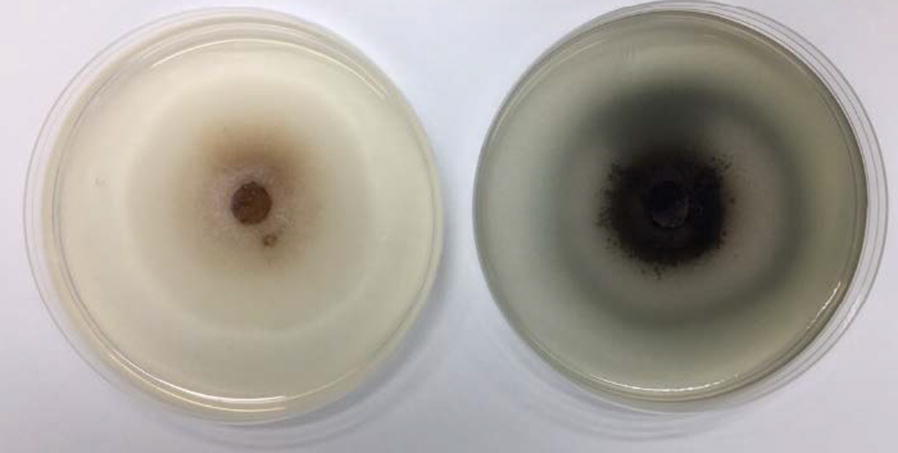
*Chaetomella raphigera* D2 strain secretes a β-glucosidase. *C. raphigera* was cultured for 4 days at 25 °C on PDA plates with (right) or without (left) β-glucoside esculin. Compared to the control plate (left), β-glucosidase activity is revealed by the presence of black precipitates in the PDA plate (right) supplied with 1 g/L esculin and 0.3 g/L ferric citrate as substrates

In a previous study, our research team found that the *C. raphigera* D2 fungal strain secreted a high-efficiency β-glucosidase, D2-BGL [22]. This enzyme displayed high synergism with the *T. reesei* cellulase Celluclast 1.5L (C1.5L). The cellulase mixture containing D2-BGL exhibited a cellulose degradation efficiency close to that of a mixture supplemented with commercial *Aspergillus* β-glucosidase Novozyme 188 (N188). Here, we found that D2-BGL activity was readily detectable in culture medium, evidenced by the formation of a dark-colored complex upon hydrolysis of the β-glucoside esculin in PDA plates (Fig. 1). Phylogenetic analysis revealed that fungal β-glucosidases including D2-BGL are distinct from yeast and bacterial GH3 β-glucosidases (Additional file 1: Figure S1 and Table S1). D2-BGL has 72% amino acid sequence similarity with the GH3 protein from the necrotrophic fungus *Botrytis cinerea*, and lower than 42% similarity with the β-glucosidase AaBGL1 from *Aspergillus aculeatus*. Given its low sequence similarity to other reported GH3 enzymes, D2-BGL might have potentially new and interesting structural and functional features to be explored.

**Fig. 2:**
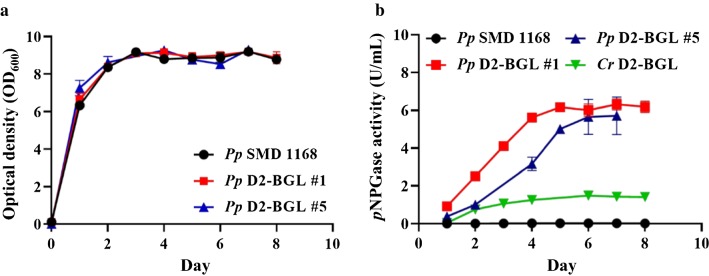
Heterologous expression of D2-BGL in *Pichia pastoris*. **a** *Pichia pastoris* wild-type and the D2-BGL-expressing Pp D2-BGL #1 and #5 strains have similar growth curves. **b** Production yield of the β-glucosidase D2-BGL is fourfold higher in yeast *Pp* D2-BGL #1 and #5 strains than in the native fungus (*Cr* D2-BGL)

The coding sequence of D2-BGL was transformed into *P. pastoris* strain SMD1168 for constitutive expression. In the flask culture, growth curves were similar between *Pp* D2-BGL #1, *Pp* D2-BGL #5 and the wild-type strain SMD1168 (Fig. 2a). Maximum β-glucosidase activity (6 U/mL) was reached 6 days after inoculation for both of the D2-BGL-expressing strains, i.e., #1 and #5 (Fig. 2b). Enzyme production was fourfold higher in *Pp* D2-BGL #1 than in the native fungus (6.2 vs 1.4 U/mL), but the purification yield was only 5.4% (Additional file 1: Table S2). Addition of an extra 6-histidine tag in *Pp* D2-BGL #5 effectively increased the purification yield from 5.4% to 30.9%, but codon optimization did not improve recombinant protein production. Native and recombinant enzymes did not exhibit significant endo-glucanase, exo-glucanase or xylanase activities (Additional file 1: Table S3).

Improved enzyme production through expression in *P. pastoris* represents the first step to evaluate D2-BGL as a potential β-glucosidase supplement in the *T. reesei* RUT-C30 cellulase preparation used for industrial applications. The *T. reesei* RUT-C30 mutant is widely used as a cellulase producer because of its ability to hypersecrete soluble proteins, including a large proportion of endo-glucanases and exo-glucanases, resulting in its cellulose hydrolysis efficiency being almost threefold greater than that of the wild-type strain [34]. However, without addition of exogenous β-glucosidases in the cellulase preparation, its capacity for cellulose hydrolysis is limited because it lacks substantial cellobiase activity and due to the effects of product inhibition on cellulases from cellobiose and glucose [35]. Accordingly, combining recombinant D2-BGL with *T. reesei* RUT-C30 cellulases could increase the efficiency of enzymatic hydrolysis of lignocellulosic biomass to an industrial scale.

### Characterization and kinetics of D2-BGL

To compare the catalytic efficiency of D2-BGL with the commercial β-glucosidase N188, we purified both enzymatic and performed kinetic analyses (Additional file 1: Table S4). We observed two major peaks on the chromatogram when we purified D2-BGL by IMAC. The *p*NPGase activity assay indicated that eluted fractions from the second peak at 30% elution buffer showed higher enzyme activity than fractions from the first peak (Additional file 1: Figure S2a). SDS-PAGE analysis suggests that D2-BGL is present in most of the second peak (Additional file 1: Figure S2b). However, proteins from the second peak generated a smeared band of apparent molecular weights much greater than the expected size of D2-BGL, indicating that *P. pastoris*-expressed D2-BGL may be hyperglycosylated. The crude preparation of N188 showed four major protein bands with molecular weights estimated as 140, 120, 72 and 60 kDa when analyzed by SDS-PAGE (Additional file 1: Figure S3a). An initial purification was performed by anion-exchange chromatography to remove the 140-kDa protein, and the 120-kDa protein was separated from the other two proteins by size-exclusion chromatography. *p*NPGase activity assay confirmed that only the 120-kDa protein had β-glucosidase activity (fractions 18 to 20, Additional file 1: Figure S3b).

**Fig. 3:**
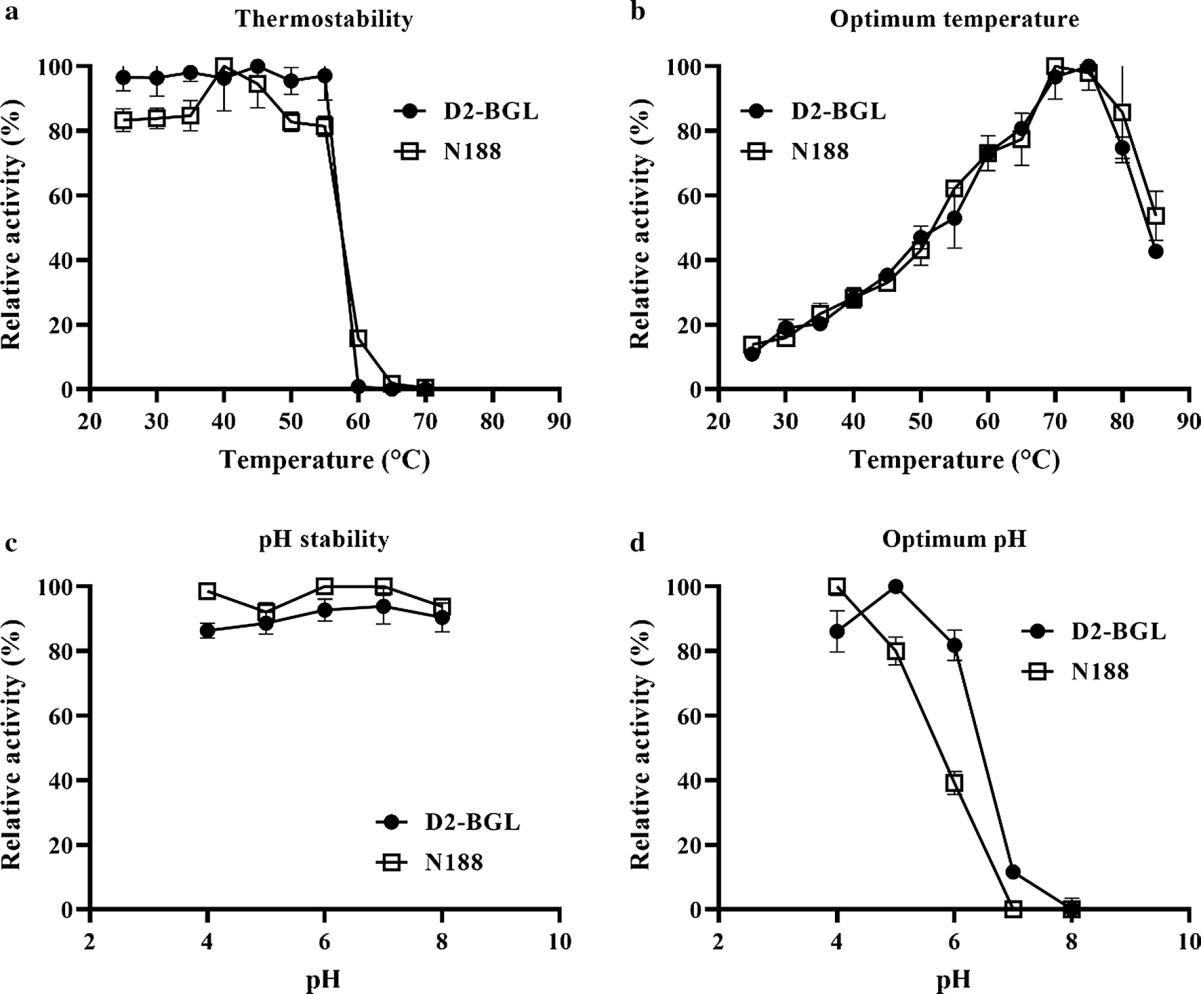
Effects of temperature and pH on the β-glucosidases D2-BGL and Novozyme 188 (N188). D2-BGL and N188 retained more than 80% of enzyme activity after 4-h incubation at 55 °C (**a**), and the optimal temperature was 70 °C (**b**). Relative activity remained > 80% at pH 4–8 after 24-h incubation at 4 °C (**c**), and the optimal pH was < 5 (**d**). Enzyme assays were performed in triplicate with *p*NPG as substrate, and error bars represent the standard deviation

We characterized the effects of temperature and pH on the activities of purified D2-BGL and N188. Both of these β-glucosidases retained more than 80% activity after 4-h incubation at 55 °C (Fig. 3a). Relative activity greatly diminished at temperatures > 55 °C, with D2-BGL being more thermolabile than N188. The optimal temperature for enzymatic activity of both enzymes was 70 °C (Fig. 3b). D2-BGL and N188 both remained stable within pH 4–8, maintaining > 80% relative activity (Fig. 3c). The optimal pH was 5 for D2-BGL and 4 for N188 (Fig. 3d), suggesting that these β-glucosidases have better catalytic efficiency in acidic environments than in neutral or slightly basic (pH 8) environments.

Kinetic parameters were determined using *p*NPG and cellobiose as substrates (Table 1 and Additional file 1: Figure S4). *K*_m_ values were tenfold lower for D2-BGL than N188 for *p*NPG (0.2 vs 2.14 mM) and more than twofold lower for cellobiose (0.96 vs 2.38 mM), so D2-BGL has higher substrate affinity than N188. However, *V*_max_ values were lower for D2-BGL than N188 for *p*NPG (419 vs 886 U/mg) and cellobiose (313 vs 1471 U/mg). The effect of substrate inhibition was observed at 1 mM for D2-BGL and at 4 mM for N188 when *p*NPG was used as a substrate (Additional file 1: Figure S4c), which suggests that the high substrate affinity of D2-BGL is coupled with a low tolerance to substrate inhibition. The *K*_i_ values were 2.42 ± 0.69 for D2-BGL and 4.68 ± 0.35 for N188, which suggests that D2-BGL is less tolerant to product inhibition than N188.

**Table 1:** Kinetic parameters for D2-BGL and Novozyme 188

Substrate	*K*_m_ (mM)	*V*_max_ (U/mg)	*K*_i_ glucose (mM)
D2-BGL			
*p*NPG	0.2 ± 0.1	419 ± 59	2.42 ± 0.69
Cellobiose	0.96 ± 0.26	313 ± 20	
N188			
*p*NPG	2.14 ± 1.18	886 ± 300	4.68 ± 0.35
Cellobiose	2.38 ± 1.38	1471 ± 383	

*Pichia pastoris* has been used to heterologously express cellulolytic enzymes from the fungi *Periconia sp.*, *Aspergillus fumigatus* Z5 strain, *Myceliophthora thermophila*, *Penicillium funiculosum* NCL1 strain, *Neurospora crassa* and *Talaromyce leycettanus*, with *K*_m_ values ranging from 0.18 to 2.5 mM for *p*NPG and 0.5 to 10.4 mM for cellobiose (Table 2) [14, 15, 17, 36–39]. Hence, D2-BGL expressed in *P. pastoris* has relatively high substrate affinity (i.e., low *K*_m_). We suggest that the dynamic synergism between D2-BGL and *T. reesei* cellulases we observed previously [22] is due to the efficient hydrolysis ability of D2-BGL despite low cellobiose concentration generated during the early phase of cellulose saccharification.

**Table 2:** Comparison of kinetic parameters between D2-BGL and other *Pichia pastoris*-expressed fungal β-glucosidases

Organism	*p*NPGase activity		Cellobiase activity		References
	*K*_m_ (mM)	*V*_max_ (U/mg)	*K*_m_ (mM)	*V*_max_ (U/mg)	
*A. fumigatus* Z5	1.76	131	2.2	53	[15]
*M. thermophila*	0.39	47.9	2.64	49.4	[37]
*N. crassa*	0.21	147.9	ND	75	[38]
*P. funiculosum* NCL1	2.5	3332	1.25	2083	[17]
*Periconia sp*. BCC2871	0.19	761	0.5	627	[36]
*T. leycettanus* JCM12802	0.18	1309	10.4	618	[39]
*T. leycettanus*	1.1	469	7.6	526	[14]
*C. raphigera* D2	0.2	419	0.96	312.5	This study

*ND* not determined

### A cellulase preparation containing D2-BGL efficiently hydrolyzes acid-pretreated biomass

**Fig. 4:**
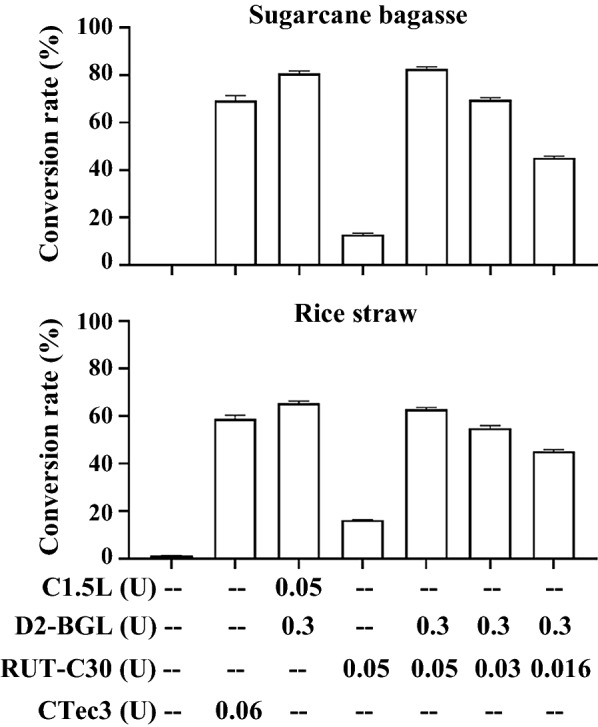
Cellulase mixtures complemented with the β-glucosidase D2-BGL efficiently hydrolyze acid-pretreated sugarcane bagasse and rice straw. Enzyme amounts are represented by exo-glucanase activity units (for Avicel substrate) for C1.5L, RUT-C30 and CTec3 and by β-glucosidase activity (for *p*NPG substrate) for D2-BGL. The conversion rate is defined as the total weight of glucose obtained after hydrolysis divided by the total weight of cellulose in the biomass. Experiments were performed in triplicate, and error bars represent the standard deviation

We evaluated the efficiency of D2-BGL as a β-glucosidase supplement for lignocellulosic biomass hydrolysis. Two cellulase mixtures were prepared by adding D2-BGL to the commercial product C1.5L (C1.5L + D2) or to a laboratory-generated fermentation broth of the *T. reesei* RUT-C30 strain (RUT-C30 + D2) (Fig. 4). We employed the commercial cellulase preparation CTec3 containing all three types of cellulases as a positive control for the hydrolysis assay. Cellulase activities of CTec3, C1.5L and RUT-C30 cellulase preparations are presented in Additional file 1: Table S5. We found that 70% of cellulose was converted into glucose from acid-pretreated sugarcane bagasse using 0.06 exo-glucanase unit (equivalent to 6.7 FPU per gram of biomass) of CTec3. The conversion rate was 80% for the cellulase mixture of 0.05 exo-glucanase unit (equivalent to 5.1 FPU per gram of biomass) of C1.5L and 0.3 β-glucosidase unit of D2-BGL. When 0.05 exo-glucanase unit (equivalent to 27.8 FPU per gram of biomass) of RUT-C30 was used alone, the conversion rate was 13%, but this outcome was considerably increased to 54%, 70% and 83% upon the addition of D2-BGL to 0.016, 0.03 and 0.05 exo-glucanase unit of RUT-C30, respectively, which suggests that the addition of D2-BGL could solve the lack of β-glucosidase in *T. reesei* cellulases and economize the amount of cellulases used in biomass hydrolysis. Furthermore, conversion rates were 60%, 65% and 63% during rice straw hydrolysis for CTec3, C1.5L + D2 and RUT-C30 + D2 preparations, respectively.

Several *P. pastoris*-expressed fungal β-glucosidases have been reported for their efficiency as enzyme supplements in the C1.5L *T. reesei* cellulase mixture for lignocellulosic biomass hydrolysis. For example, upon addition of recombinant *Periconia sp.* BGL 1, reducing sugar released from rice straws pretreated by steam explosion was increased by 70%, and the use of recombinant *Humicola insolens* Y1 HiBGL3C released 27.67 mM of reducing sugar (including 84% of glucose) from NaOH-pretreated corn stover [36, 40]. Saccharification of wheat straw slurry was enhanced using *Pichia*-expressed *Talaromyces amestolkia*e BGL-3 relative to the commercial β-glucosidase N188 (37% vs 17%) [41]. In this study, we observed that the formulation of 0.3 unit of D2-BGL with 0.05 unit of exo-glucanase from *T. reesei* RUT-C30 cellulase was the optimal cellulase ratio for hydrolyzing 1% (w/v) sugarcane bagasse powder. We also observed that the commercial CTec3 has higher β-glucosidase to FPase ratio than other cellulase preparations (Additional file 1: Table S6). Considering that CTec3 has been used without additional supplements for the industrial-scale saccharification with high biomass concentrations to yield high levels of glucose, we speculate that the addition of seemingly excessive amounts of β-glucosidase in this commercial cellulase mixture is probably needed to overcome the negative effects of substrate and product inhibitions on β-glucosidase. Therefore, further investigations are necessary to optimize the compositional ratio of D2-BGL to *T. reesei* cellulases for efficient hydrolysis of high concentrations of biomass.

### A cellulase preparation containing D2-BGL accelerates ethanol production during SSSF

**Fig. 5:**
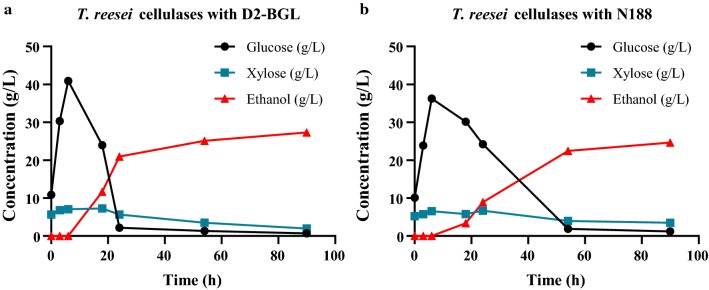
Use of the β-glucosidase D2-BGL accelerates ethanol production during a semi-simultaneous saccharification and fermentation process. A *T. reesei* cellulase mixture was combined with D2-BGL (**a**) or N188 (**b**) to hydrolyze acid-treated rice straws in a 100-L bioreactor. Upon adding *S. cerevisiae* at the 12-h time point, ethanol was produced faster by the cellulase mixture containing D2-BGL than by the one hosting N188

The SSSF process comprised a pre-saccharification phase during the first 12 h with a progressively increased glucose concentration due to rice straw hydrolysis, followed by a simultaneous saccharification and fermentation phase with ethanol production coupled with glucose consumption by *S. cerevisiae*. Using the cellulase preparation containing D2-BGL as a β-glucosidase supplement (Fig. 5a), the glucose concentration reached 40 g/L after 6 h. Twelve hours after yeast inoculation (i.e., 24 h from the beginning of the SSSF process), the ethanol concentration reached 21 g/L and the glucose concentration was ~ 2 g/L. Thereafter, most of the cellulose in rice straws was digested by cellulases, resulting in slight changes in both ethanol and glucose concentrations. When N188 was used as a β-glucosidase supplement, ethanol production was 8.9 g/L and 22.4 g/L at 24 and 54 h from commencement of the SSSF process, respectively (Fig. 5b). Xylose concentrations remained similar throughout the process in these two experiments. These results indicate that the time needed to produce the same amount of ethanol was halved when D2-BGL rather than N188 was used in the SSSF process.

### Upscaling production of D2-BGL to a 1-ton bioreactor

**Fig. 6:**
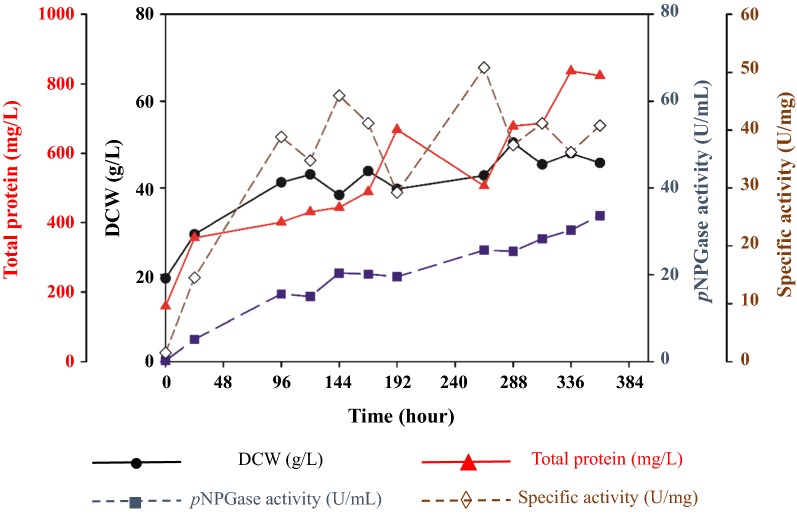
β-Glucosidase activity and yeast titers during D2-BGL production in a 1-ton bioreactor. DCW: dry cell weight

Since D2-BGL has the potential to be used for biomass saccharification at an industrial level, we developed a fermentation procedure for D2-BGL production in a 1-ton bioreactor. Two sequential stages of fermentation were required to produce the high cell density culture of *P. pastoris* for the 1-ton fermentation process. Dry cell weight (DCW) values were about 100 g/L in a 5-L bioreactor and about 70 g/L in a 100-L bioreactor at the end of each stage. In the 1-ton bioreactor, the glycerol used as the sole carbon source in the fermentation medium was almost completely consumed after 24 h (Additional file 1: Figure S5). Dissolved oxygen (DO) decreased rapidly until 18 h, but then increased markedly from 2 to 36% at 24 h, indicating complete consumption of glycerol at that time point and with a rapid increase of cell density to 29 g/L (Fig. 6). Feeding started after 24 h, and all fed glycerol was totally used by the end of the fermentation process. Yeast biomass increased by 56% from the 24-h time point to the end of fermentation (29.3–45.75 g/L). Since D2-BGL is produced continuously using the constitutive glyceraldehyde-3-phosphate promoter (pGAP), *p*NPGase activity increased progressively during fermentation until maximum enzyme activity of 33.6 U/mL was reached on day 15, equivalent to the productivity of 0.1 g/L of recombinant enzyme.

Expression of most of the reported *Pichia*-expressed β-glucosidases is regulated by the methanol-inducible alcohol oxidase 1 (AOX1) promoter. However, storage and feeding of methanol require a specific factory design and equipment to avoid fire hazards during industrial fermentation [42]. Here, we propose using the strong constitutive GAP promoter to produce D2-BGL, and we intend to further explore optimizations of the culture medium recipe to improve productivity.

### D2-BGL crystal structure analysis reveals a three-domain β-glucosidase with specific glycosylation sites

**Fig. 7:**
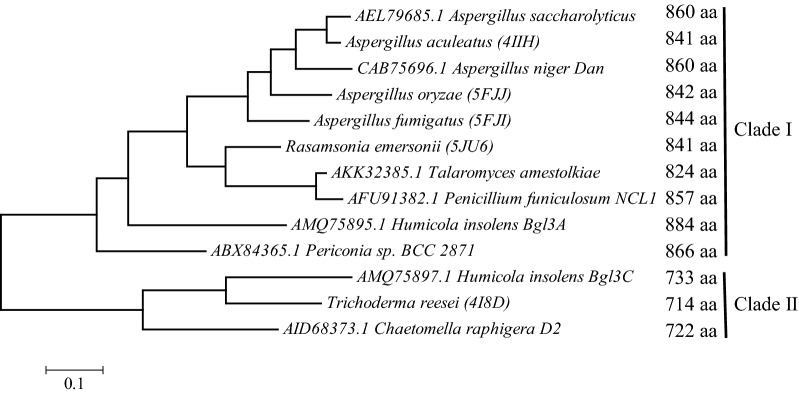
Phylogenetic analysis of fungal GH3 β-glucosidases showing two enzyme clades differing in protein lengths. D2-BGL is a clade II enzyme with fewer than 800 amino acid residues

**Fig. 8:**
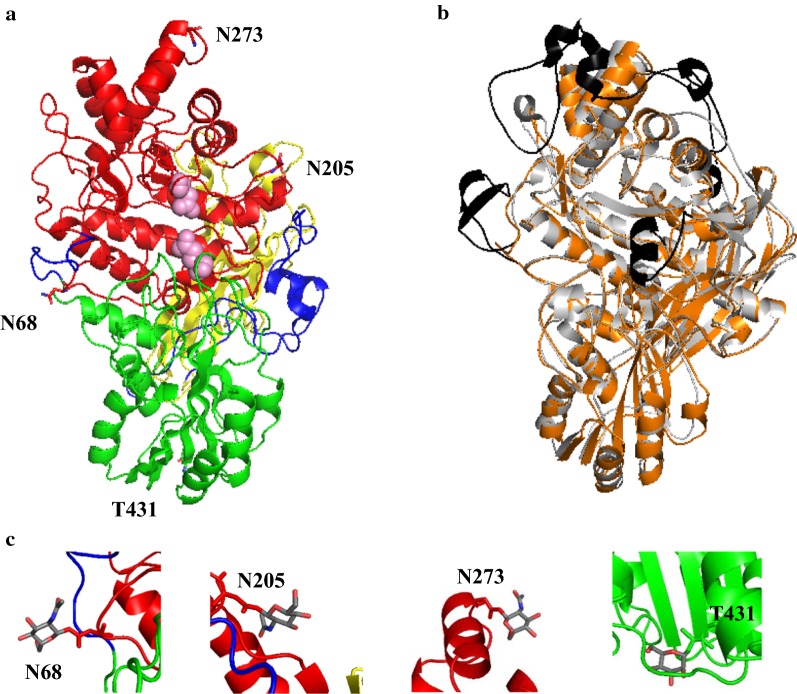
Crystal structure analysis indicates that D2-BGL is a GH3 β-glucosidase with specific glycosylation sites. **a** β-Glucosidase D2-BGL consists of a TIM barrel-like domain (in red), an α/β sandwich domain (in green), and a fibronectin type III-like domain (in yellow). D232 and E442, which are represented by pink spheres, indicate the location of the catalytic center. **b** Structure superposition reveals that three extra insertion domains (in black) observed in the *Aspergillus aculeatus* β-glucosidase AaBGL1 (in grey) are absent from D2-BGL (in orange). **c** Glycosylation sites are determined by the presence of N-acetyl glucosamine at residues N68, N205 and N273 for *N*-glycosylation and by a mannose at T431 for *O*-glycosylation. N68 and T431 glycosylation have not been observed in any other reported GH3 β-glucosidases

Based on our phylogenetic study, we separated fungal β-glucosidases into two clades. *A. aculeatus* (PDB entry: 4IIH [18]), *A. oryzae* (PDB entry: 5FJJ [20]), *A. fumigatus* (PDB entry: 5FJI [20]) and *Rasamsonia emersonii* (PDB entry: 5JU6 [21]) β-glucosidases belong to clade I enzymes with more than 824 amino acid residues, and *T. reesei* Cel3A (PDB entry: 4I8D [19]) and D2-BGL belong to clade II enzymes with less than 733 amino acid residues (Fig. 7). As D2-BGL has a protein sequence similarity of < 60% with these other β-glucosidases, we investigated whether it also has specific structural features potentially involved in substrate binding or protein stability. For this purpose, we determined the 3D structure of D2-BGL by X-ray crystallography at 1.9-Å resolution (accession code 6JXG). Data collection and final model statistics are presented in Additional file 1: Table S7. D2-BGL has the classical three-domain structure of a GH3 β-glucosidase: a TIM barrel-like domain (residues 1–307), an α/β sandwich domain (residues 319–521), and a fibronectin type III-like domain (residues 573–712) (Fig. 8a). There are three intra-domain disulfide bonds involving residues C39–C55, C199–C210 and C369–C374. Three *N*-glycosylation sites (N68, N205 and N273) were determined by the presence of *N*-acetylglucosamine after endoglycosidase H (Endo H) treatment, and one O-linked mannose was observed on residue T431 (Fig. 8c).

**Fig. 9:**
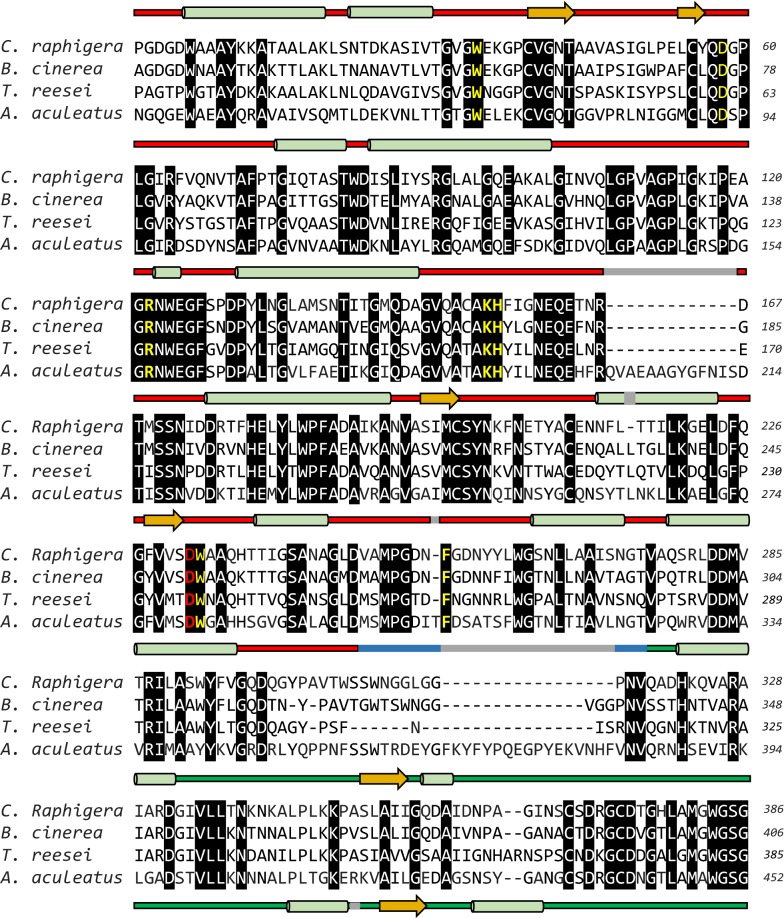

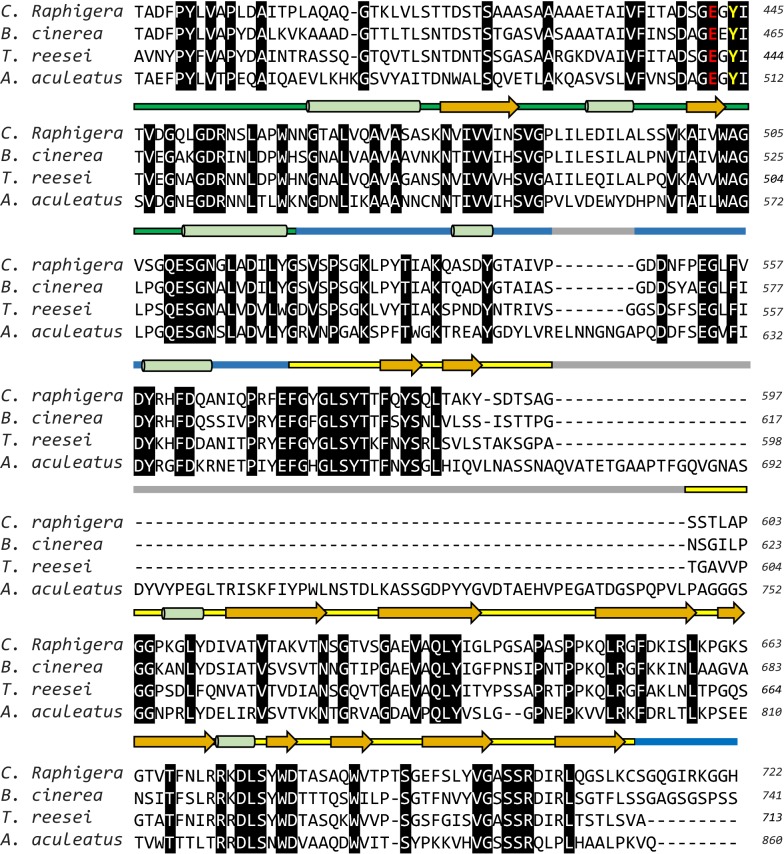
Multiple sequence alignment reveals structural diversity between clade I and II β-glucosidases. Three insertion regions, that are observed in *Aspergillus aculeatus* AaBGL1 (clade I), are not found in *C. raphigera* D2-BGL (clade II). The “prominent insertion region” observed in *Aspergillus aculeatus* AaBGL1 is absent from D2-BGL (from residues 597 to 603). The loop connecting TIM barrel and α/β sandwich domains are shorter in D2-BGL (from residues 307 to 318). D2-BGL has a shorter loop (from residues 166 to 168) than the corresponding loop in AaBGL1 at the entry region of the active site in the TIM barrel-like domain (from residues 200 to 215). In the multiple sequence alignment, amino acid residues involved in substrate binding and in catalysis reaction are in yellow and red, respectively. In the schema of the 3D structure of D2-BGL, TIM barrel-like domain, α/β sandwich domain and fibronectin III-like domain are represented by red, green and yellow segments, respectively. Gray segments represent sequences observed in AaBGL1. Green cylinder: α-helix; orange arrow: β-sheet

To elucidate the unique features of D2-BGL relative to clade I β-glucosidases, we performed structure alignment between D2-BGL and *A. aculeatus* BGL1 (AaBGL1, PDB: 4IIH) (Fig. 8b). The root mean square deviation (RMSD) of 615 pairs of Cα atoms was 0.9 Å. In addition, multiple sequence alignment revealed three insertion regions in AaBGL1 comparing to D2-BGL, *B. cinerea* GH3 protein and *T. reesei* β-glucosidase Cel3A (Fig. 9). The “prominent insertion region”—observed in the fibronectin III-like domain of AaBGL1 (between amino acids a.a. 671 and 747) and in β-glucosidases from *R. emersonii*, *A. oryzae* and *A. fumigatus*—is absent from D2-BGL. The connection loop between the TIM barrel-like domain and the α/β sandwich is shorter in D2-BGL than in AaBGL1 (12 a.a. from residues 307 to 318 vs 29 a.a. from residues 356 to 384). This loop plays a role in forming protein dimer in AaBGL1 [18], but we did not observe that dimerization state for the D2-BGL crystal structure. Another short loop is close to the active site entry region in D2-BGL (3 a.a. from residues 166 to 168), which may influence the efficiency of enzyme catalysis by broadening the active site pocket. These results indicate that clade II fungal enzymes such as D2-BGL or *T. reesei* β-glucosidase Cel3A are functional β-glucosidases with a more compact protein structure.

We also determined the key amino acid residues involved in the active site of D2-BGL by structure alignment with AaBGL1. In D2-BGL, residues D232 and E442 are nucleophile and general acid/base residues, respectively. D58, R122, K155, H156 and W233 form the substrate-binding subsite −1. The hydrophobic substrate-binding subsite +1 is formed by W34, Y444 and F256. Structure alignment revealed that all key amino acid residues lie in the same positions for both D2-BGL and AaBGL1, except for the phenylalanine involved in substrate-binding subsite +1 (i.e., F256 in D2-BGL and F305 in AaBGL1), in which the two aromatic rings have different orientations. The N68 and T431 glycosylation sites have not been observed in crystal structures of other reported GH3 β-glucosidases. Given that glycosylation plays roles in protein stability and substrate-binding residues mediate enzymatic catalysis, we further examined the functions of these two intriguing features in D2-BGL.

### Presence of specific glycosylation sites is essential for producing recombinant D2-BGL

**Fig. 10:**
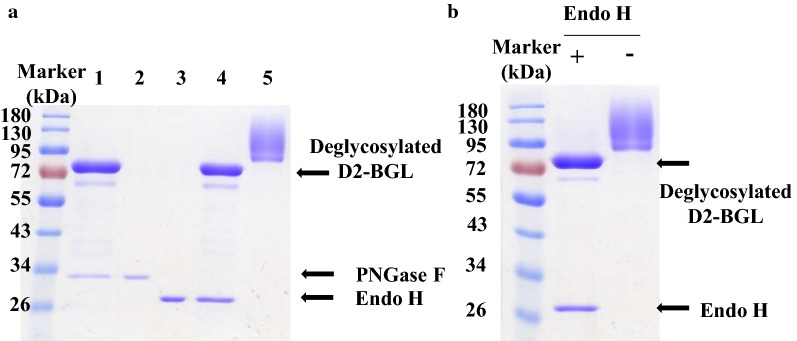
*Pichia pastoris*-expressed recombinant D2-BGL exhibits variable hypermannosylation. **a** Yeast-expressed D2-BGL is highly mannosylated (line 5). Peptide N-glycosidase F (PNGase F, lines 1 and 2) and endo-glucosidase H (Endo H, lines 3 and 4) can remove N-glycans from *Pichia pastoris*-expressed D2-BGL under denaturing conditions (heating at 100 °C for 10 min) (lines 1 and 4). **b** N-Glycans were removed by endoglycosidase H (Endo H) treatment at 37 °C for 4 h

Our SDS-PAGE revealed a smeared band of *P. pastoris*-expressed D2-BGL ranging from 95 to 180 kDa, which suggests that it may be hyperglycosylated to varying degrees. To determine the type of D2-BGL glycosylation, we carried out enzymatic deglycosylation using peptide N-glycosidase F (PNGase F) and Endo H. Only one band instead of a smear was observed on SDS-PAGE under denaturing conditions upon either Endo H or PNGase F treatment, but only Endo H removed all N-glycans under non-denaturing conditions (Fig. 10 and Additional file 1: Figure S6). To evaluate the effects of hypermannosylation on catalytic activity, we examined the cellobiase activity of deglycosylated D2-BGL. Deglycosylated D2-BGL exhibited specific activity (185 ± 21 U/mg) close to that of non-deglycosylated enzyme (209 ± 14 U/mg), indicating that the presence of N-glycans does not affect the catalytic activity of D2-BGL. In addition, we created D2-BGL mutants by site-directed mutagenesis in which N68 was substituted by glutamine (N68Q) and T431 was replaced by alanine (T431A) or serine (T431S) (Table 3). Altering these glycosylation sites reduced the enzyme activity measured in the culture supernatant of the N68Q mutant (0.98 ± 0.22 U/mL) and T431A mutant (0.61 ± 0.05 U/mL) relative to that of wild-type (1.89 ± 0.18 U/mL). The T431S mutant (in which the O-glycosylation site T431 was substituted by S) showed similar enzyme activity (1.89 ± 0.03 U/mL) to wild type. However, specific activity of purified enzyme was similar for all four strains (197 ± 3, 216 ± 23, 189 ± 11 and 225 ± 20 U/mg for wild type, N68Q, T431A and T431S, respectively), resulting in lower enzyme productivity for the N68Q and T431A mutant strains than for wild type (4.5 and 3.2 vs 9.6 mg/L, respectively).

**Table 3:** Comparison of enzyme activity and production between wild-type D2-BGL and the N68Q, T431A and T431S mutants

	Activity (U/mL)	Specific activity (U/mg)	Productivity (mg/L)
Wild type	1.89 ± 0.18	197 ± 3	9.6
N68Q	0.98 ± 0.22	216 ± 23	4.5
T431A	0.61 ± 0.05	189 ± 11	3.2
T431S	1.89 ± 0.03	225 ± 20	8.4

Glycosylation plays a variety of roles in *P. pastoris*-expressed fungal enzymes. For example, N-glycosylation is essential for proper protein folding and the secretion of recombinant *Aspergillus terreus* β-glucosidase [43], whereas O-glycosylation decreases pH stability of *T. leycettanus* β-glucosidase [39]. In our previous study [27], we observed that *C. raphigera*-expressed native D2-BGL has two glycosylation variants, and the large-form native D2-BGL with greater O-glycosylation showed higher enzyme activity toward cellobiose. For *P. pastoris*-expressed D2-BGL, both N-glycosylation and O-glycosylation may function in enzyme stability and secretion. Heterologous expression of β-glucosidases in *Saccharomyces cerevisiae* has been considered a solution in industrial applications for reducing the inhibitory effect of cellobiose on endo-glucanases and exo-glucanases during the hydrolysis of lignocellulosic biomass in SSSF [44]. However, *S. cerevisiae* exhibits protein hyperglycosylation that occasionally reduces the catalytic efficiency of recombinant cellulases originating from filamentous fungi [45]. Due to its tolerance of hyperglycosylation, D2-BGL may represent a potential candidate for heterologous expression in *S. cerevisiae* for SSSF processes.

### High substrate affinity of D2-BGL is due to the position of F256 in a short loop near the substrate-binding site

**Fig. 11:**
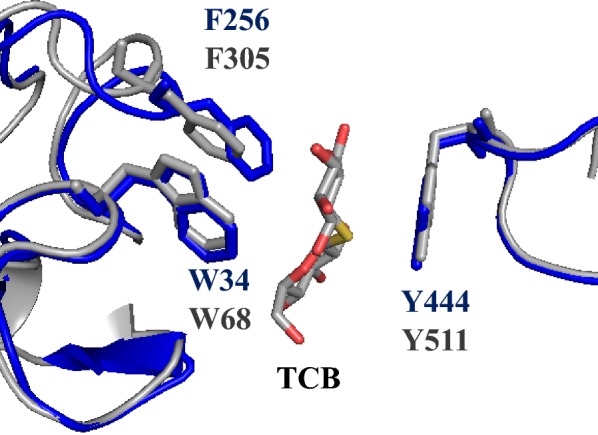
Substrate-binding residue F256 presents a specific orientation in D2-BGL relative to that in AaBGL1. The W–F–Y triad (in stick form) acts as substrate-binding subsite +1 in D2-BGL (in blue) and in *Aspergillus aculeatus* β-glucosidase AaBGL1 (in grey). *TCB* thiocellobiose

**Fig. 12:**
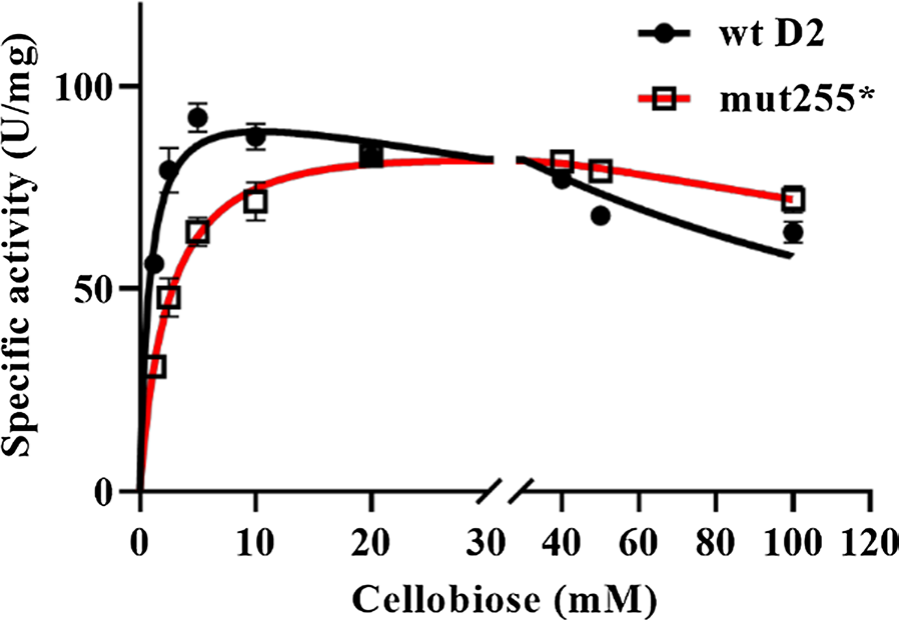
Substrate affinity toward cellobiose is decreased in the N255* mutant. Mutant N255* has lower substrate affinity than wild-type D2-BGL, which results in lower enzyme activity at low cellobiose concentrations. Enzyme assays were performed in triplicate, and error bars represent the standard deviation

During protein sequence and structure analyses, we observed that the orientation of the aromatic ring in the substrate-binding residue F256 of D2-BGL differed from that of homologous F305 in AaBGL1 (Fig. 11). In addition, all clade II β-glucosidases have a loop near F256 or equivalent that is one amino acid shorter than the same loop in clade I enzymes (Additional file 1: Figure S7a). To clarify the relationship between F256 position, loop length and substrate affinity, we generated several mutants at residues N255 and G257 by site-directed mutagenesis. Mutants N255D, N255S and G257D were designed by consensus mutagenesis between D2-BGL and *Aspergillus* species enzymes. The activities of these mutants were decreased at different concentrations of cellobiose without an obvious decrease in substrate affinity, indicating that the amino acid composition at positions N255 and G257 essentially controls catalytic efficiency (Additional file 1: Figure S7b). To determine whether substrate affinity is controlled by loop length, we created the N255* mutant in which N255 was substituted by two amino acid residues (IS) to make the loop one amino acid longer. The specific activity of the crude N255* mutant enzyme was greatly reduced at cellobiose contents of < 20 mM, but it was slightly higher than wild-type activity at cellobiose concentrations > 20 mM (Fig. 12). *K*_m_ values were 1.45 mM for wild-type D2-BGL and 2.84 mM for mutant N255*, which suggests that the short loop is crucial for the high substrate affinity of D2-BGL.

For most fungal GH3 β-glucosidases, the triad of hydrophobic amino acids W–F–Y is responsible for the binding of the non-reducing sugar moiety of cellobiose or other β-glycosides at the substrate-binding subsite +1. These residues are W34, F256 and Y444 in D2-BGL, corresponding to W68, F305 and Y511 in AaBGL1 and W37, F260 and Y443 in *Hypocrea jecorina* HjCel3A [18, 19]. The shorter loop of D2-BGL probably makes it less flexible, which favors the T-shaped conformation between the aromatic rings of W34 and F256. The consequences for substrate affinity may also differ between clade I and clade II β-glucosidases when mutations occur close to substrate-binding residues in the substrate-binding subsite +1.

## Conclusions

Fungal β-glucosidases are widely used for their highly efficient cellobiose hydrolysis during saccharification of lignocellulosic biomass. We heterologously expressed β-glucosidase D2-BGL (isolated from the fungus *C. raphigera*) in *P. pastoris* using the strong constitutive GAP promoter. Recombinant D2-BGL showed higher substrate affinity than *A. niger* β-glucosidase Novozyme 188 and, when used as a supplement in a *T. reesei* cellulase mixture, efficiently hydrolyzed acid-pretreated rice straws and sugarcane bagasse. Use of D2-BGL also accelerated ethanol production via a semi-simultaneous saccharification and fermentation process. Our 3D crystal structure analysis revealed that D2-BGL has specific *N*- and *O*-glycosylation sites indispensable for enzyme production. Discovery of the specific orientation of F256 in D2-BGL provides new insights into substrate binding in GH3 β-glucosidases. We also successfully upscaled enzyme production in a 1-ton bioreactor, making it suitable for industrial applications.

## Supplementary information

**Additional file 1: Figure S1.** Phylogenetic analysis of microbial GH3 β-glucosidases. The closest sequence to D2-BGL, i.e. *B. cinerea* GH3 protein, exhibits only 72% sequence similarity to D2-BGL. Among filamentous fungi, Ascomycota phylum is highlighted in yellow and Basidiomycota phylum is highlighted in orange.** Table S1.** Accession number of GH3 β-glucosidases mentioned in the phylogenetic tree.** Table S2.** Summary of D2-BGL sequence modifications and their effect on recombinant protein productivity and purification. T1572C: silent mutation. M1-9: codon modification from CGC to AGA at positions M1:364–366, M2:496–498, M3:838-840, M4:859–861, M5:979–981, M6:1114–1116, M7:1360–1362, M8:1951–1953 and M9:2011–2013.** Table S3.** Activity of native D2-BGL and *P. pastoris*-expressed D2-BGL toward different substrates.** Table S4.** Purification tables of D2-BGL and Novozyme 188 (N188).** Figure S2.** Purification of D2-BGL by affinity chromatography. (a) The chromatograph shows that the major part of recombinant D2-BGL was eluted with 30% of elution buffer. b) SDS-PAGE analysis suggests that most D2-BGL is found in the second peak.** Figure S3.** Sequential purification of N188. (a) Partially purified N188 was found in elution fractions 21 to 23 after anion-exchange chromatography. (b) After site-exclusion chromatography, purified N188 was collected from elution fractions 18 and 19.** Figure S4.** Kinetics of D2-BGL and N188. Determination of *K*_m_ and *V*_max_ using cellobiose (a and b) or *p*NPG (c and d) as substrates, and determination of inhibition constant *K*_i_ using *p*NPG as substrate for D2-BGL (e) and for N188 (f).** Table S5.** Cellulase activities of different enzyme mixtures used during the experiment of biomass saccharification. **Table S6.** Cellulase activities in enzyme mixtures used for sugarcane bagasse saccharification.** Figure S5.** Variation of glycerol and ammonia concentrations during fermentation in a one-ton bioreactor. **Table S7.** D2-BGL crystallographic data collection and refinement statistics.** Figure S6.** PNGase F cannot remove N-glycans without denaturation at 100 °C. Line 1: D2-BGL with PNGase F, line 2: D2-BGL without PNGase F.** Figure S7.** Mutations at N255 and G257 cause loss of enzyme activity. (a) Among all amino acid residues involved in the active site pocket, N255, F256 and G257 differ between D2-BGL and β-glucosidases from *Aspergillus*, resulting in D2-BGL having a shorter loop. (b) Point mutation at position N255 (mutants N255D and N255S) or G257 (mutant G257D) reduces the cellobiase activity of D2-BGL.

## Data Availability

The datasets used and/or analyzed during the current study are available from the corresponding author on reasonable request.

## Abbreviations

*p*NPG: *p*-nitrophenyl β-d-glucopyranoside
CMC: carboxymethylcellulose
DNS: dinitrosalicylic acid
