# Supplementary material for: Chaetomella raphigera β-glucosidase D2-BGL has intriguing structural features and a high substrate affinity that renders it an efficient cellulase supplement for lignocellulosic biomass hydrolysis
Source: Biotechnol Biofuels. 2019 Nov 2;12:258. doi: 10.1186/s13068-019-1599-0 (PMC6825360; doi:10.1186/s13068-019-1599-0)
Supplement: Supplementary file 1 — Additional file 1: Figure S1. Phylogenetic analysis of microbial GH3 β-glucosidases. The closest sequence to D2-BGL, i.e. B. cinerea GH3 protein, exhibits only 72% sequence similarity to D2-BGL. Among filamentous fungi, Ascomycota phylum is highlighted in yellow and Basidiomycota phylum is highlighted in orange. Table S1. Accession number of GH3 β-glucosidases mentioned in the phylogenetic tree. Table S2. Summary of D2-BGL sequence modifications and their effect on recombinant protein productivity and purification. T1572C: silent mutation. M1-9: codon modification from CGC to AGA at positions M1:364–366, M2:496–498, M3:838-840, M4:859–861, M5:979–981, M6:1114–1116, M7:1360–1362, M8:1951–1953 and M9:2011–2013. Table S3. Activity of native D2-BGL and P. pastoris-expressed D2-BGL toward different substrates. Table S4. Purification tables of D2-BGL and Novozyme 188 (N188). Figure S2. Purification of D2-BGL by affinity chromatography. (a) The chromatograph shows that the major part of recombinant D2-BGL was eluted with 30% of elution buffer. b) SDS-PAGE analysis suggests that most D2-BGL is found in the second peak. Figure S3. Sequential purification of N188. (a) Partially purified N188 was found in elution fractions 21 to 23 after anion-exchange chromatography. (b) After site-exclusion chromatography, purified N188 was collected from elution fractions 18 and 19. Figure S4. Kinetics of D2-BGL and N188. Determination of Km and Vmax using cellobiose (a and b) or pNPG (c and d) as substrates, and determination of inhibition constant Ki using pNPG as substrate for D2-BGL (e) and for N188 (f). Table S5. Cellulase activities of different enzyme mixtures used during the experiment of biomass saccharification. Table S6. Cellulase activities in enzyme mixtures used for sugarcane bagasse saccharification. Figure S5. Variation of glycerol and ammonia concentrations during fermentation in a one-ton bioreactor. Table S7. D2-BGL crystallographic data collection and refinement statisti [file 13068_2019_1599_MOESM1_ESM.pdf]

Additional file 1:
Figure S1

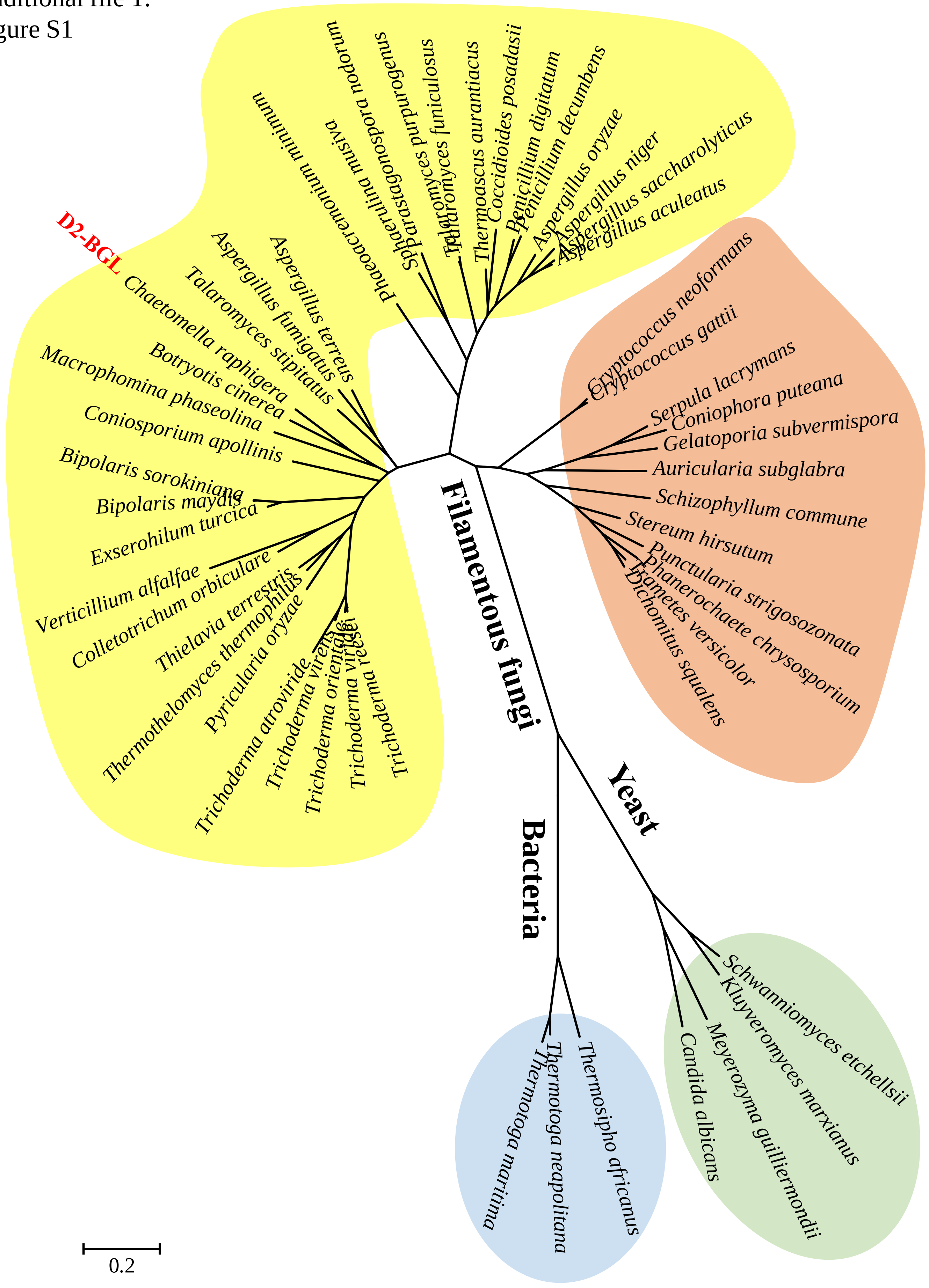

Figure S1: Phylogenetic analysis of microbial GH3 β-glucosidases. The closest sequence to D2-BGL, i.e. *B. cinerea* GH3 protein, exhibits only 72% sequence similarity to D2-BGL. Among filamentous fungi, Ascomycota phylum is highlighted in yellow and Basidiomycota phylum is highlighted in orange.

Additional file 1:
Table S1

Table S1: Accession number of GH3 β-glucosidases mentioned in the phylogenetic tree.

Fungal species	Accesion number
Aspergillus aculeatus	BAA10968.1
Aspergillus fumigatus	XP_748896.1
Aspergillus niger	CBA02054.1
Aspergillus oryzae	XP_001816831.1
Aspergillus saccharolyticus	AEL79685.1
Aspergillus terreus	XP_001216552.1
Auricularia subglabra	EJD46362.1
Bipolaris maydis	EMD92964.1
Bipolaris sorokiniana	XP_007700786.1
Botrytis cinerea	CCD47324.1
Candida albicans	EEQ44248.1
Chaetomella raphigera	AID68373.1
Coccidioides posadasii	EFW20210.1
Colletotrichum orbiculare	TDZ19988.1
Coniophora puteana	XP_007774882.1
Coniosporium apollinis	XP_007780683.1
Cryptococcus gattii	XP_003192746.1
Cryptococcus neoformans	XP_569544.1
Dichomitus squalens	XP_007360087.1
Exserohilum turcica	XP_008028859.1
Gelatoporia subvermispora	EMD32780.1
Kluyveromyces marxianus	XP_022675159.1
Macrophomina phaseolina	EKG15351.1
Meyerozyma guilliermondii	XP_001484535.1
Parastagonospora nodorum	AAT95384.1
Penicillium decumbens	ADB82653.1
Penicillium digitatum	XP_014531524.1
Phaeoacremonium minimum	XP_007917209.1
Phanerochaete chrysosporium	BAB85988.1
Punctularia strigosozonata	XP_007378452.1
Pyricularia oryzae	XP_003720272.1

Schizophyllum commune	XP_003037081.1
Schwanniomyces etchellsii	ACF93471.1
Serpula lacrymans	EGO02892.1
Sphaerulina musiva	XP_016765664.1
Stereum hirsutum	XP_007307502.1
Talaromyces funiculosus	AFU91382.1
Talaromyces marneffei	XP_002149046.1
Talaromyces purpureogenus	ACV87737.1
Talaromyces stipitatus	XP_002485128.1
Thermoascus aurantiacus	AFU51372.1
Thermothelomyces thermophilus	XP_003663588.1
Thielavia terrestris	XP_003653677.1
Trichoderma atroviride	XP_013940584.1
Trichoderma orientale	AFM77966.1
Trichoderma reesei	XP_006964076.1
Trichoderma virens	XP_013957182.1
Trichoderma viride	AAQ76093.1
Verticillium alfalfae	XP_003001548.1

Additional file 1:
Table S2

Table S2: Summary of D2-BGL sequence modifications and their effect on recombinant protein productivity and purification. T1572C: silent mutation. M1-9: codon modification from CGC to AGA at positions M1:364-366, M2:496-498, M3:838-840, M4:859-861, M5:979-981, M6:1114-1116, M7:1360-1362, M8:1951-1953 and M9:2011-2013.

Clone	Sequence modification	Sequence scheme	Activity (U/mL)	Purification yield (%)
D2-BGL	Native	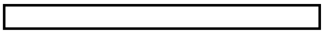	1.4 ± 0.2	--
Pp D2-BGL #1	T1572C	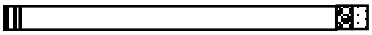	6.2 ± 0.4	5.4
Pp D2-BGL #5	M1-9/T1572C/N-His	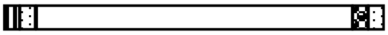	6.0 ± 1.0	30.9

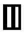: α-factor secretory peptide 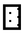: His-tag 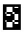: myc epitope

Additional file 1:
Table S3

Table S3: Activity of native D2-BGL and *P. pastoris*-expressed D2-BGL toward different substrates.

	Activity (U/mg[a])				
**Substrate**	***Cr* D2-BGL**	***Pp* SMD 1168**	***Pp* D2-BGL #1**	***Pp* D2-BGL #5**	**N188**
1% CMC[b]	2.5 ± 0.3	1.4 ± 0.1	1.4 ± 0.1	1.9 ± 0.2	0.1 ± 0.1
1% Xylan	0.2 ± 0.2	< 0.1	0.1 ± 0.1	0.1 ± 0.1	5.0 ± 0.9
1% Avicel	< 0.1	< 0.1	< 0.1	< 0.1	< 0.1
FP[c]	< 0.1	< 0.1	< 0.1	< 0.1	< 0.1

a: total crude enzyme, b: carboxymethylcellulose, c: filter paper

Additional file 1:
Table S4

Table S4: Purification tables of D2-BGL and Novozyme 188 (N188).

D2-BGL								
**Fraction**	**Volume (mL)**	**Activity (U/mL)**	**Protein concentration (mg/mL)**	**Total activity (U)**	**Total protein (mg)**	**Specific activity (U/mg)**	**Purification fold**	**Yield (%)**
Culture supernatant	1000	69	ND	69000	ND	ND	ND	100%
Filtration	970	41.6	0.42	40352	407.4	99	0	58%
Buffer exchange	370	77.8	0.7	28786	259	111.1	1.1	42%
Ni column	92.5	185.1	0.59	17122	54.6	313.7	3.2	25%

Novozyme 188 (N188)								
**Fraction**	**Volume (mL)**	**Activity (U/mL)**	**Protein concentration (mg/mL)**	**Total activity (U)**	**Total protein (mg)**	**Specific activity (U/mg)**	**Purification fold**	**Yield (%)**
Culture supernatant	1	306.8	ND	306.8	ND	ND	ND	100%
Buffer exchange	50	1.9	0.13	95	6.5	14.6	0	31%
Anion-exchange	20	3.6	0.07	72	1.4	51.4	3.5	23%
Size-exclusion	4	8	0.026	32	0.104	307.7	21.1	10%

1 U is 1 μmol pNP produced per minute; ND: not determined

Additional file 1:
Figure S2

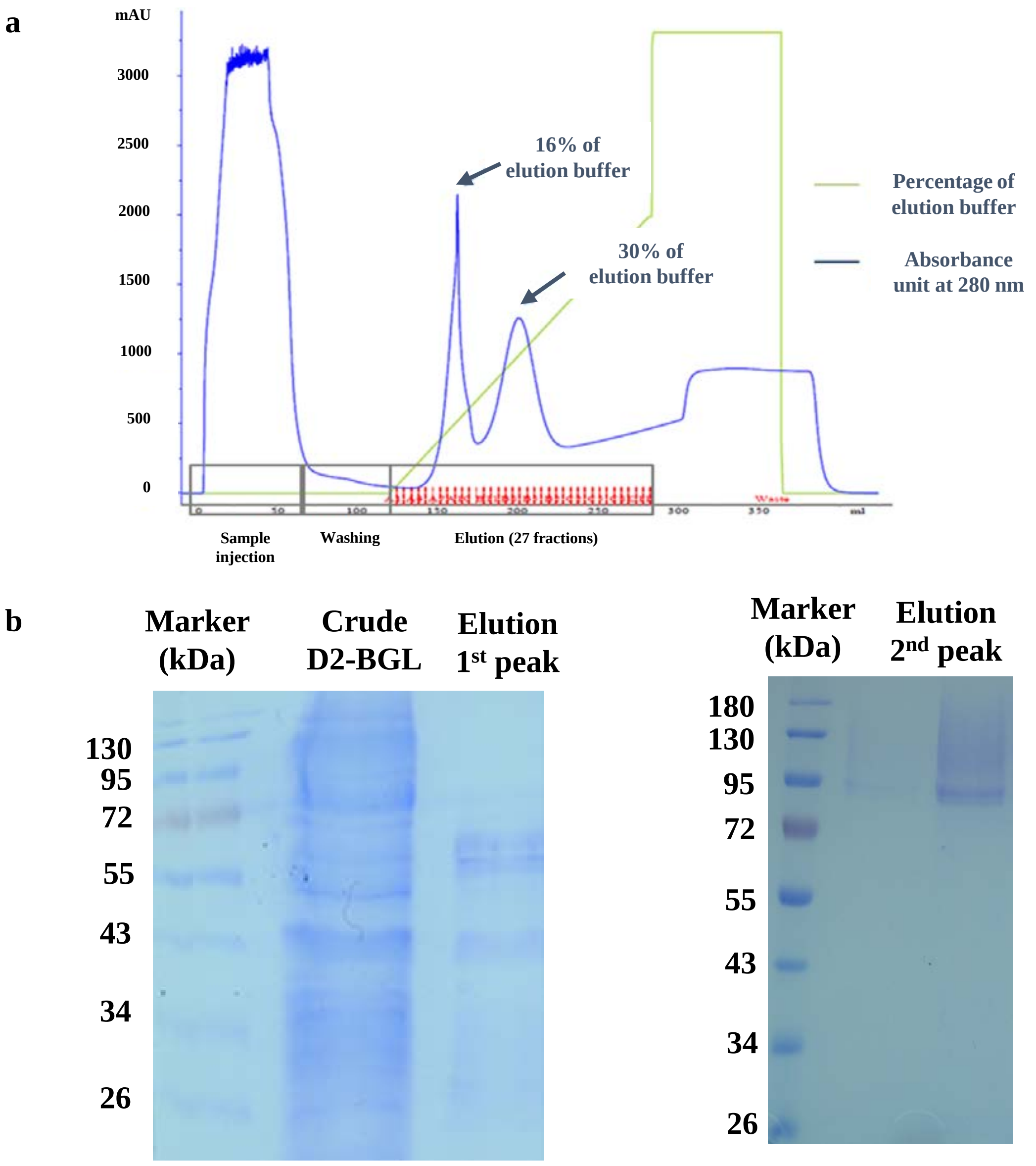

Figure S2: Purification of D2-BGL by affinity chromatography. (a) The chromatograph shows that the major part of recombinant D2-BGL was eluted with 30% of elution buffer. b) SDS-PAGE analysis suggests that most of D2-BGL is found in the second peak.

Additional file 1:
Figure S3

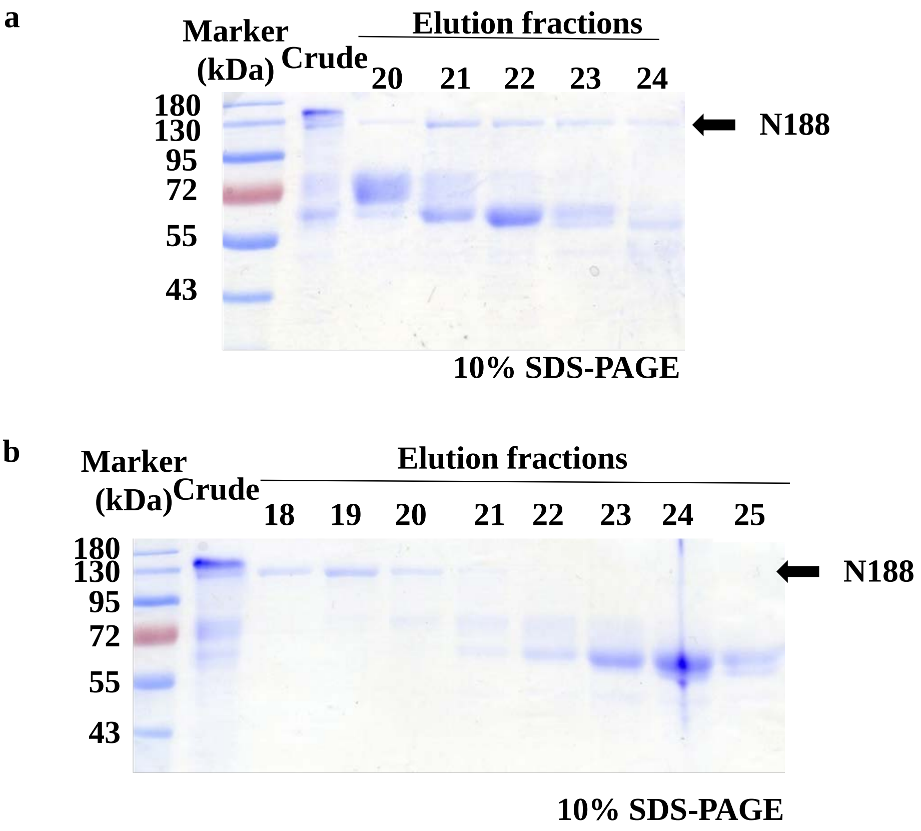

Figure S3: Sequential purification of N188. (a) Partially purified N188 was found in elution fractions 21 to 23 after anion-exchange chromatography. (b) After site-exclusion chromatography, purified N188 was collected from elution fractions 18 and 19.

Additional file 1:
Figure S4

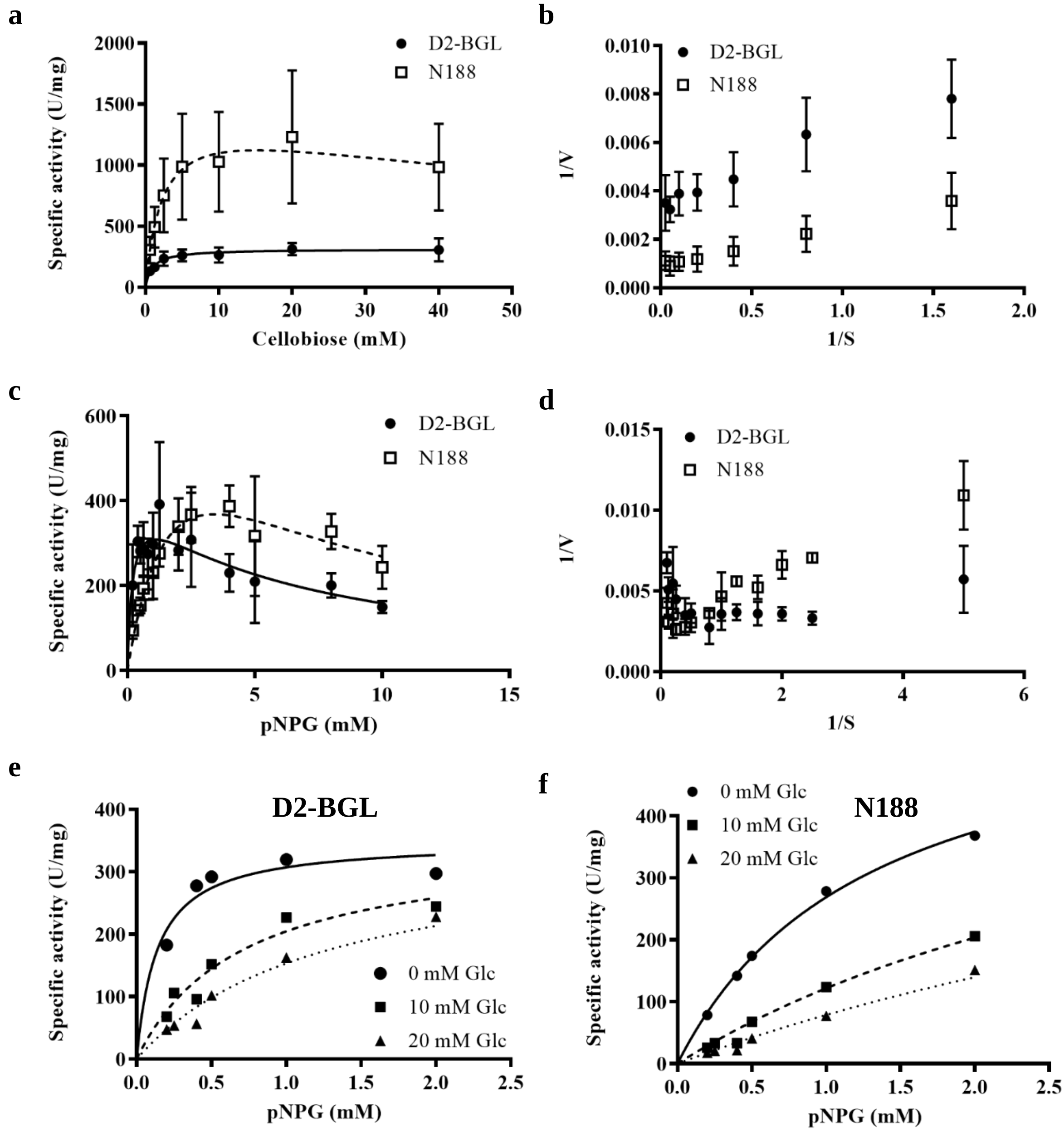

Figure S4: Kinetics of D2-BGL and N188. Determination of K_m and V_{max} using cellobiose (a and b) or *p*NPG (c and d) as substrates, and determination of inhibition constant K_i using *p*NPG as substrate for D2-BGL (e) and for N188 (f).

Additional file 1:
Table S5

Table S5: Cellulase activities of different enzyme mixtures used during the experiment of biomass saccharification.

Enzyme preparation	Cellulase activity (U/ml)		
	**β-glucosidase (*p* NPG)**	**Exoglucanase (Avicel)**	**Total cellulase activity (FP)**
**CTec3**	11000	132	148
**C1.5L**	3.1	54.5	56.1
**RUT-C30**	1.4	3.7	20.6

Additional file 1:
Table S6

Table S6: Cellulase activities in enzyme mixtures used for sugarcane bagasse saccharification.

**Cellulase preparation**	**Cellulase activity (U)**		**Conversion rate (%)**
	**β-glucosidase**	**FPase**	
CTec3 (0.06 U)*	5	0.07	69.4
C1.5L(0.05 U)* + D2-BGL (0.3 U)**	0.3	0.05	80.9
RUT-C30 (0.05 U)*	0.02	0.28	13.0
RUT-C30 (0.05 U)* + D2-BGL (0.3 U)**	0.02+0.3	0.28	82.8
RUT-C30 (0.03 U)* + D2-BGL (0.3 U)**	0.02+0.3	0.17	69.7
RUT-C30 (0.016 U)* + D2-BGL (0.3 U)**	0.02+0.3	0.09	45.2

* Exoglucanase unit

** β-glucosidase unit

Additional file 1:
Figure S5

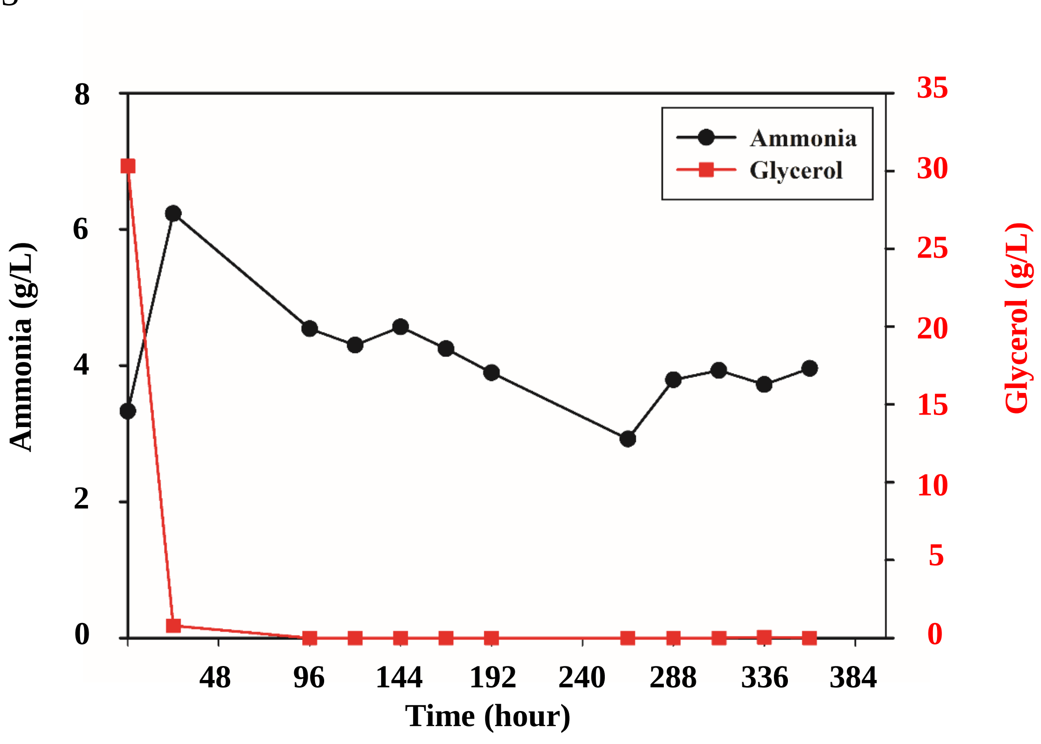

Figure S5: Variation of glycerol and ammonia concentrations during fermentation in a one-ton bioreactor.

Additional file 1:
Table S7

Table S7: D2-BGL crystallographic data collection and refinement statistics.

**Data collection**	
Wavelength (Å)	1.000
Space group	$P2_12_12_1$
Cell dimensions (Å)	*a* =67.77, *b* = 77.66, *c*=123.48
Resolution (Å)	30.00-1.90 (1.97-1.90)
Unique reflections	49,111
R_{merge} (%)	9.0 (47.5)
$I/\sigma(I)$	27.7 (6.4)
Completeness	93.8 (91.5)
Redundancy	8.0 (8.2)
**Refinement**	
Resolution (Å)	30.00-1.90
No. of reflections R_{work}/R_{free}	46,547/2,504
R_{work}/R_{free}	14.1/19.3
No. of atoms/Avg B factor (Å^2)	
Protein	5,277/16.4
Water	527/23.5
Glycan	53/30.4
RMSD	
Bond lengths (Å)	0.019
Bond angles (°)	1.98
Ramachandran statistics (%)[b]	
Ramachandran favored	96.2
Ramachandran outliers	0.28

[a] Values corresponding to the highest resolution shell are shown in parentheses.

[b] The stereochemistry of the model was validated with MolProbity [1]

1. Chen, V.B., et al., *MolProbity: all-atom structure validation for macromolecular crystallography.* Acta crystallographica. Section D, Biological crystallography, 2010. **66**(Pt 1): p. 12-21.

Additional file 1:
Figure S6

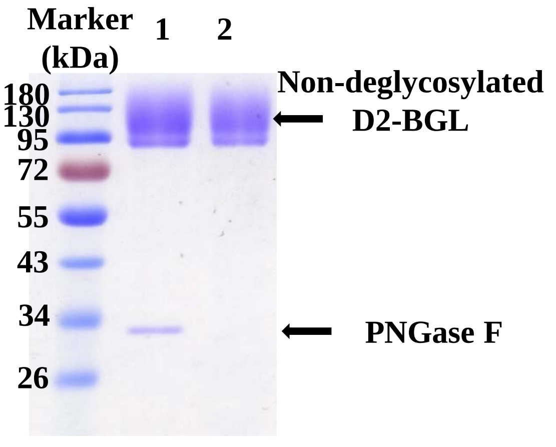

Figure S6: PNGase F cannot remove N-glycans without denaturation at 100°C. Line 1: D2-BGL with PNGase F, line 2: D2-BGL without PNGase F.

Additional file 1:
Figure S7

a

```
Aspergillus_fumigatus_(5FJI)               282 PGDISFDDGL 291
Aspergillus_oryzae_(5FJJ)                  282 PGDVTFDSGT 291
CAB75696.1_Aspergillus_niger_Dan           300 PGDVDYDSGT 309
AEL79685.1_Aspergillus_saccharolyticus     300 PGDVSFDSAT 309
Aspergillus_aculeatus_(4IIH)               300 PGDITFDSAT 309
Trichoderma_reesei_(4I8D)                  257 PGT-DFNGNN 265
AID68373.1_Chaetomella_raphigera_D2        252 PGD-NFGDNY 260
```

b

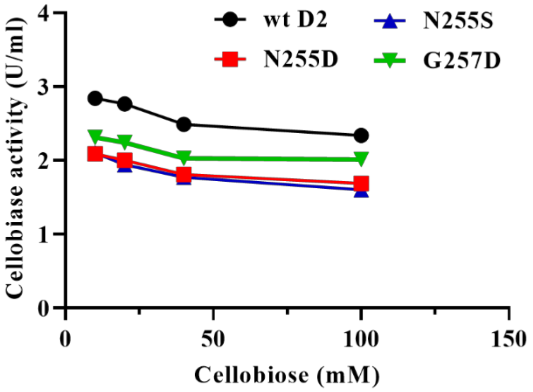

Figure S7: Mutations at N255 and G257 cause loss of enzyme activity. (a) Among all amino acid residues involved in the active site pocket, N255, F256 and G257 differ between D2-BGL and β-glucosidases from *Aspergillus*, resulting in D2-BGL having a shorter loop. (b) Point mutation at position N255 (mutants N255D and N255S) or G257 (mutant G257D) reduces the cellobiase activity of D2-BGL.
